# LOV Takes a Pick: Thermodynamic and Structural Aspects of the Flavin-LOV-Interaction of the Blue-Light Sensitive Photoreceptor YtvA from *Bacillus subtilis*

**DOI:** 10.1371/journal.pone.0081268

**Published:** 2013-11-21

**Authors:** Matthias Dorn, Marcel Jurk, Anne Wartenberg, Aaron Hahn, Peter Schmieder

**Affiliations:** 1 Department of Structural Biology, Leibniz-Institut für Molekulare Pharmakologie, Berlin, Germany; 2 Department of Chemistry and Biochemistry, Freie Universität Berlin, Berlin, Germany; Ohio State University, United States of America

## Abstract

LOV domains act as versatile photochromic switches servicing multiple effector domains in a variety of blue light sensing photoreceptors abundant in a multitude of organisms from all kingdoms of life. The perception of light is realized by a flavin chromophore that upon illumination reversibly switches from the non-covalently bound dark-state to a covalently linked flavin-LOV adduct. It is usually assumed that most LOV domains preferably bind FMN, but heterologous expression frequently results in the incorporation of all natural occurring flavins, i.e. riboflavin, FMN and FAD. Over recent years, the structures, photochemical properties, activation mechanisms and physiological functions of a multitude of LOV proteins have been studied intensively, but little is known about its affinities to physiologically relevant flavins or the thermodynamics of the flavin-LOV interaction. We have investigated the interaction of the LOV domain of the well characterized bacterial photoreceptor YtvA with riboflavin, FMN and FAD by ITC experiments providing binding constants and thermodynamic profiles of these interactions. For this purpose, we have developed a protocol for the production of the apo forms of YtvA and its isolated LOV domain and we demonstrate that the latter can be used as a molecular probe for free flavins in cell lysates. Furthermore, we show here using NMR spectroscopic techniques and Analytical Ultracentrifugation that the flavin moiety stabilizes the conformation of the LOV domain and that dimerization of YtvA is caused not only by intermolecular LOV-LOV but also by STAS-STAS contacts.

## Introduction

Photoreceptor proteins are known to regulate important cellular functions in plants, fungi, archaea and bacteria by converting an extra-cellular light signal into an intra-cellular response. The processes controlled in a light-dependent manner vary from transcription to movement of chloroplasts and phototaxis [1]. In a variety of blue light sensing photoreceptors flavin binding LOV (light, oxygen, voltage) and BLUF (blue light sensing using FAD) domains act as versatile photochromic switches servicing multiple effector domains [1–5].

Over recent years, the structures, photochemical properties, activation mechanisms and physiological functions of a multitude of LOV and BLUF proteins have been studied intensively [1–4,6,7], but little is known about its affinities to physiologically relevant flavins or the thermodynamics of the flavin-LOV interaction. This kind of knowledge could, however, be useful to assess the often unknown physiological chromophore composition of LOV photoreceptors in vivo. LOV domains share a common fold with the protein backbone comprising four α-helices and an anti-parallel beta-sheet with two of the four α-helices running parallel to it. The flavin chromophore is bound in a deep cleft with the isoalloxazine ring pointing towards the beta-sheet and the ribityl chain being sandwiched by the two α-helices [3]. It is assumed that BLUF domains preferably bind FAD [1,3,8,9] and most LOV domains preferably bind FMN [1,3,10–13]. Heterologous expression of both domains frequently results in mixtures of all natural occurring flavins, i.e. riboflavin (RF), FMN and FAD [3,14–19]. Hence, the flavin composition seems to depend on the expression system (i.e. bacteria, fungi or insect cells), the expression conditions (i.e. media, temperature, duration) and, finally, the characteristics of the domain itself.

The LOV domain of WC-1, for example, isolated from *Neurospora crassa* contains exclusively FAD [20], whereas the heterologously expressed LOV protein Vivid, from the same organism, binds both FAD and FMN with ratios varying from 0.5 to 1 depending on the purified batch [16]. In contrast, LOV domains of heterologously expressed *Arabidopsis thaliana* phototropin-1 and LOV1 of *Chlamydomonas reinhardtii* phototropin were found to contain exclusively FMN [11–13]. This suggests that some LOV domains can interact with different flavins, while others seem to be specific for a particular flavin type.

An inspection of several LOV structures (see Table 1) provides no general explanation why certain domains have different flavin specificities with the exception of two FAD-binding domains. The Vivid LOV domain (PDB-ID: 2PD7), despite its high similarity to the phototropin-like LOV domains, is the only known LOV structure containing FAD. It possesses an extended loop (between the two conserved α-helices mentioned above) which accommodates the adenosine moiety of FAD at the protein surface [21]. The additional polar interaction between the pyrophosphate of FAD and the side-chain of a lysine within that loop most likely enhances the affinity for FAD. This is supported by the fact, that a similar loop-extension is also present in the FAD binding LOV domain of WC-1 [20].

**Table 1:** Selection of available LOV structures.

**protein**	**PDB-ID**	**Ref.**
LOV domain of YtvA (*Bacillus subtilis*)	2PR5	[29]
LOV2 domain of Phy3 (*Adiantum capillus-veneris*)	1G28	[60]
LOV2 domain of AtPhot2 (*Arabidopsi thaliana*)	4EEP	[61]
LOV2 domain of AsPhot1 (*Avena sativa*)	2V0U	[62]
LOV1 domain of AtPhot1 (*Arabidopsis thaliana*)	2Z6C	[63]
LOV1 domain of AtPhot2 (*Arabidopsis thaliana*)	2Z6D	[63]
LOV1 domain of CrPhot1 (*Chlamydomonas reinhardtii*)	1N9L	[64]
LOV domain of LOV-HK (*Brucella abortus*)	3T50	[37]
Aureochrome1 (*Vaucheria frigida*)	3UE6	[65]
EL222 (*Erythrobacter litoralis*)	3P7N	[66]
Vivid with FAD (*Neurospora crassa*)	2PD7	[21]
Vivid with FMN (*Neurospora crassa*)	2PDT	[21]

In order to obtain information on both affinity as well as specificity of phototropin-like LOV domains for naturally occurring flavins, we have studied the interaction of the deflavinated LOV domain of the well characterized protein YtvA from *Bacillus subtilis* with RF, FMN and FAD. YtvA is involved in the general stress response pathway of the common soil bacterium *B. subtilis* [22–25] and was recently shown to be permanently associated with the bacterial stressosome [26]. It is a ~60 kDa homodimer possessing a dissociation constant (K_D_) below 300 nM [27] and each subunit consists of an N-terminal phototropin-like LOV domain and a C-terminal STAS (sulphate transporter / antisigma factor antagonist) domain linked to each other by a helical amino acid sequence, denoted Jα-helix. The protein adopts a dumbbell shape, with LOV-LOV and STAS-STAS interactions on either side [28-31]. The structure of its LOV domain (aa 21-147) was published in 2007 and it was shown that this truncated construct is also dimeric, with a K_D_ below 200 nM [29].

YtvA and its LOV domain are, upon heterologous expression in *E. coli*, predominantly associated with FMN ([32] and this work), but recently it was demonstrated, that YtvA is also able to incorporate RF, FMN, FAD and two synthetic flavin derivatives during a refolding procedure [33]. Here we present a protocol for the preparation of the apo form of both YtvA and its isolated LOV domain. Both can be completely reconstituted with different flavins and we provide thermodynamic profiles and binding constants of the interactions of the LOV domain with RF, FMN and FAD using Isothermal Titration Calorimetry (ITC). Based on data from Analytical Ultracentrifugation (AUC) and solution-state NMR spectroscopy, we show that the chromophore stabilizes the global fold and quaternary structure of the protein. Beyond that, we demonstrate that the deflavinated LOV domain can be used as a flavin-specific molecular probe for the determination of the relative flavin composition of cell lysates and we provide an explanation for the varying flavin compositions of heterologously expressed LOV proteins.

## Results and Discussion

### Protein constructs and naming conventions

For the investigation presented here two constructs of YtvA have been used, the full length protein (aa 1 – 261) which we call YtvA and an extended version of the LOV domain (aa 1 – 128) which we call YLOV. If the proteins are expressed heterologously in *E. coli* they mainly contain FMN and up to 11 % RF. The latter can be replaced with a large molar excess of FMN under native conditions (i.e. without unfolding of the protein, see Protocol S1 and Figure S1 in the supplementary material for further details) in both YtvA and YLOV. We call the proteins thus obtained E.coli-YtvA and E.coli-YLOV. Using the protocol described below it is possible to remove the chromophore and refold the protein in absence of any flavin, yielding Apo-YtvA and Apo-YLOV. Subsequent reintroduction of a RF, FMN or FAD, leads to RF-YtvA, FMN-YtvA and FAD-YtvA, respectively, similar names will be used for YLOV. A backbone NMR assignment has been obtained for both E.coli-YtvA [28] and E.coli-YLOV.

### Preparation of apoproteins

Several strategies for the chromophore exchange in flavoproteins and flavin binding BLUF and LOV domains have been presented: The chromophore release is typically based on either a lowering of pH to about 4 [10,34], sometimes combined with supplementation of high concentrations of KBr [14,35,36], or denaturation using urea or guanidinium chloride [33,37–39]. Reconstitution of the apoproteins is subsequently achieved by refolding or regeneration of physiological conditions in the presence of the desired flavins. In most cases, the intermediate apoproteins are quite unstable and therefore not suitable for further studies.

It has been shown, however, that flavins in the heterologously expressed BLUF domain of AppA from *Rhodobacter sphaeroides* are exchangeable under native conditions [15]. This is also true for YtvA and YLOV and since the flavin is exchangeable without unfolding the protein, we assumed that Apo-YtvA and Apo-YLOV are stable in solution.

Modified versions of the protocols employed by Eisenreich et al. [38] and Mansurova et al. [33] were used to remove the bound flavins quantitatively from E.coli-YtvA and E.coli-YLOV, the major difference being the concentration of urea used for protein denaturation, which was set to 6.5 M. The resulting apoproteins were subsequently refolded under reducing conditions (1 mM DTT) in absence of any flavin. Both apoproteins were soluble and absence of the typical flavin absorption within its UV-VIS spectra indicated a complete removal of the chromophore (see Figure S2 in the supplementary material).

### NMR-spectroscopy of apoproteins

**Figure 1:**
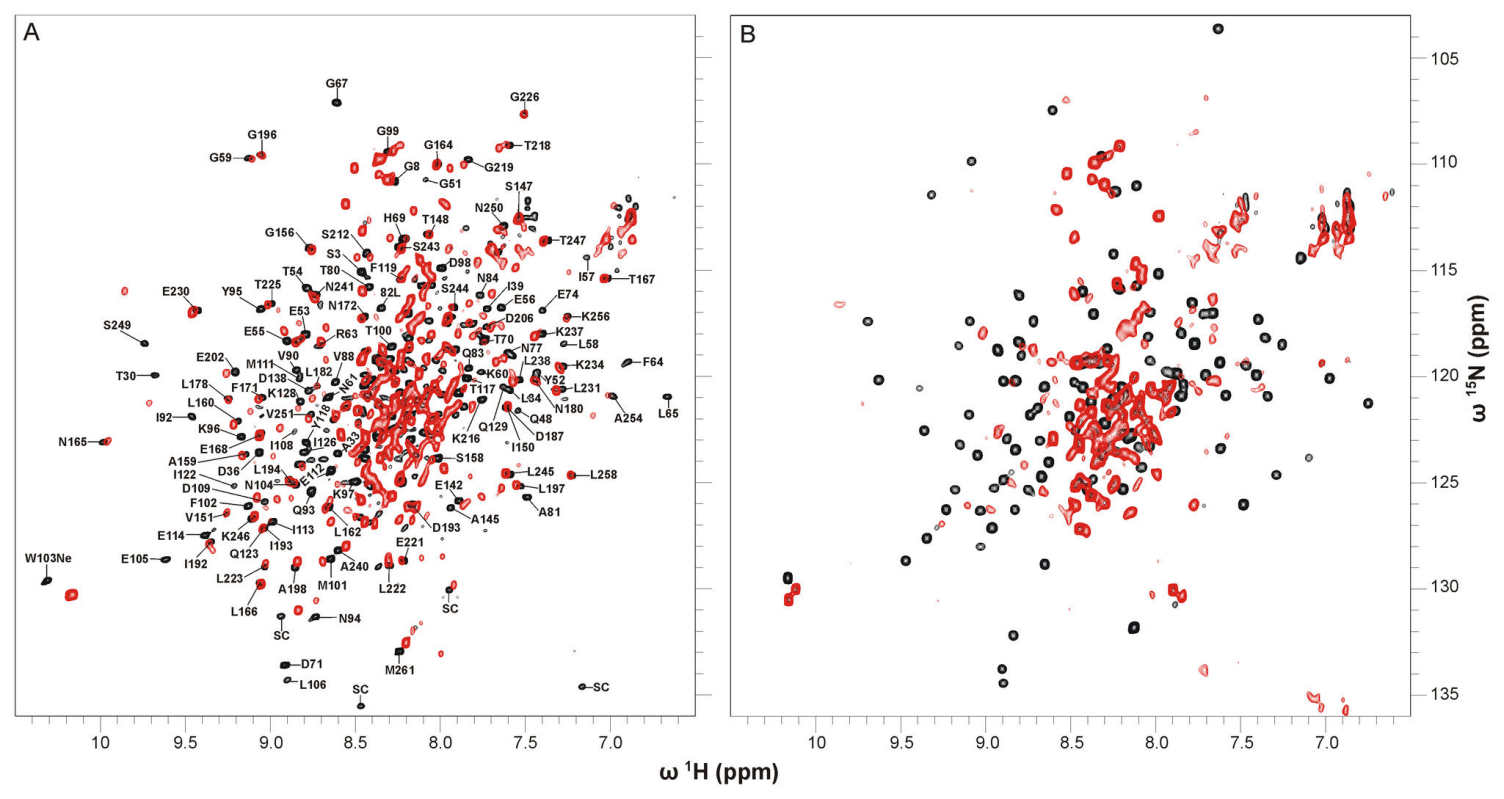
Superposition of ^1^H-^15^N-TROSY spectra of native and apo form of YtvA and YLOV. (A) ^1^H-^15^N-TROSY spectra of E.coli-YtvA (black) and Apo-YtvA (red). Both samples were uniformly ^2^H-^15^N-labeled. Sequentially assigned amide resonances of E.coli-YtvA outside the central region of the spectrum are labeled with residue type and sequence position (SC: unassigned resonances most likely belonging to side chains). (B) ^1^H-^15^N-TROSY spectra of E.coli-YLOV (black) and Apo-YLOV (red). Both samples were uniformly ^15^N-labeled.

To obtain information on the overall fold of the apoproteins two-dimensional ^1^H,^15^N correlation spectra (^1^H,^15^N-TROSYs) of uniformly ^15^N-labeled Apo-YLOV and uniformly ^2^H-,^15^N-labeled Apo-YtvA were recorded and compared with corresponding spectra of the holoproteins (Figure 1).

The spectrum of Apo-YtvA shows signal dispersion very similar to that obtained from E.coli-YtvA indicating that the apoprotein is structured and that it has a well defined overall fold. Loss of the chromophore leads, however, to shifts of various resonances. Using the resonance assignment of E.coli-YtvA [28], those resonances can be identified but since no assignment of Apo-YtvA is available, the changes in chemical shift cannot be quantified. Inspection of well resolved, non-overlapping resonances suggest that predominantly signals of amino acids of the LOV domain (aa 21 to 126) are affected, while resonances of the STAS domain (aa 148 to 261) remain largely unperturbed (Figure 1A, and enlarged in Figure S3). The absence of the flavin moiety obviously alters the chemical environment of at least 56 residues throughout the whole LOV domain. The resonance frequencies of some of these residues seem to be shifted far from its original values. Moreover, a significant number of resonances have disappeared indicating a line-broadening of the affected signals caused by a change in conformational dynamics.

Except for S^147^, all other evaluable resonances of the Jα-helix (aa 127 to 147) have disappeared, indicating that this structural element is likewise in conformational exchange. In contrast, resonances of the very C-terminal Jα-residue (S^147^) and its succeeding amino acids (T^148^ to V^151^, G^156^) are not affected or only slightly shifted suggesting that this region has a structure similar to that in E.coli-YtvA. Most of the remaining resonances of the STAS domain that can be analysed (51 out of 101 residues) are hardly affected exhibiting chemical shift differences less than the line-widths. In addition, the signal pattern resembles that in E.coli-YtvA and we therefore assume that the structure of the STAS domain is fully recovered during the refolding process.

The superposition of the ^1^H,^15^N correlation spectra of E.coli-YLOV and Apo-YLOV shows that, except for the central region, nearly all characteristic E.coli-YLOV resonances are absent or very weak for the apoprotein (Figure 1B). Analogous to Apo-YtvA this suggests that the protein is in conformational exchange, but the somewhat larger signal overlap in the central region of the spectrum could also hint at structural disorder. Assuming that Apo-YLOV and particularly its central beta-sheet were unstructured, there would be no structural basis for a dimer interface (as it is present in flavin containing YLOV [29]) which would result in monomeric Apo-YLOV. Therefore, we analysed the oligomerization state using analytical ultracentrifugation.

### Analytical Ultracentrifugation Experiments

**Figure 2:**
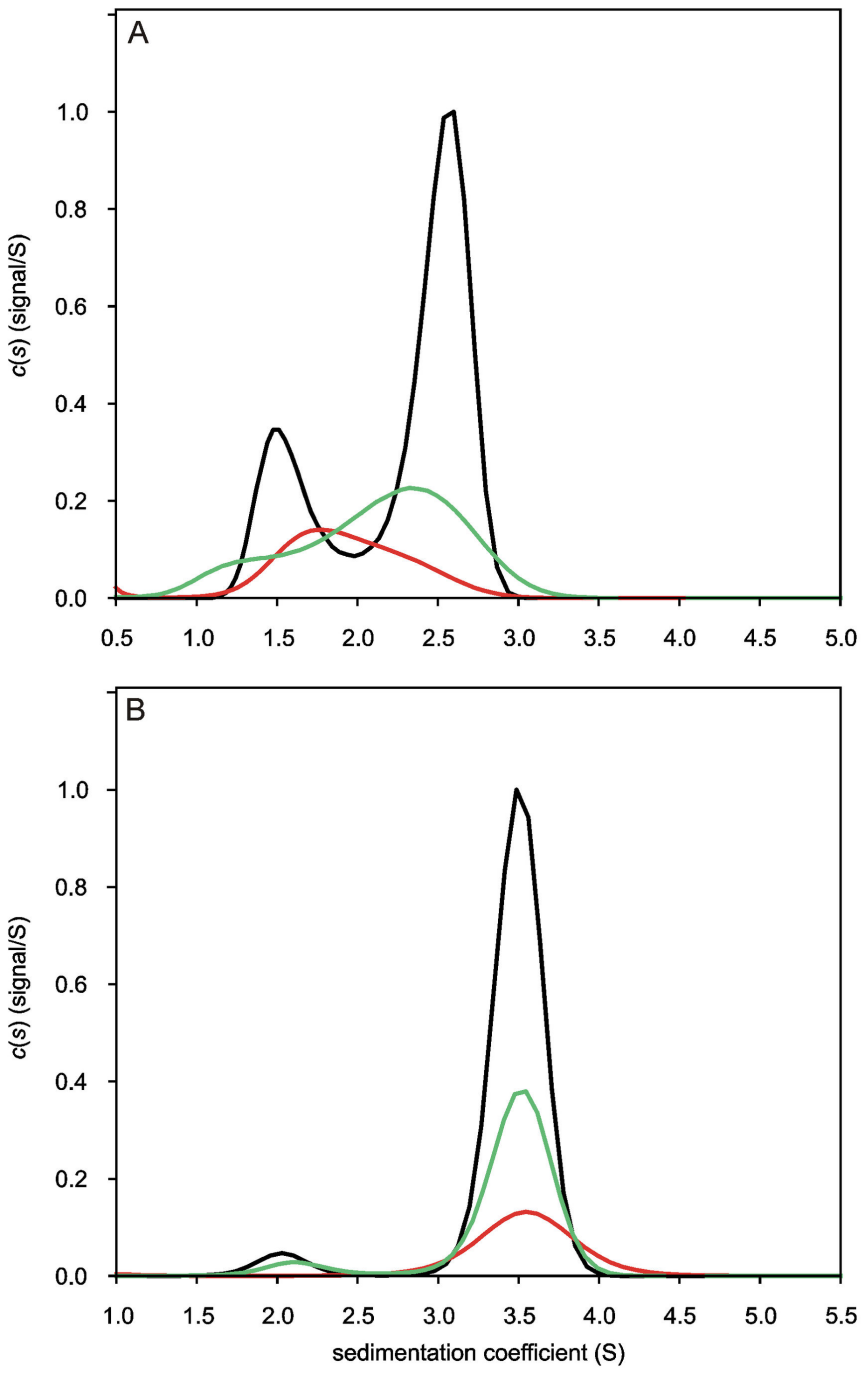
c(S)-Distributions obtained for Apo-YLOV and Apo-YtvA revealing a change in oligomerization behaviour. Sedimentation velocity (SV) experiments were performed at 45,000 rpm and sedimentation was followed at 280 nm. Maxima of c(S)-Distributions derived from highest concentration were normalized to 1 and the area of distributions from lower concentrated samples was scaled according to sample concentration. (A) Concentration dependent distribution of sedimentation coefficients derived from SV data acquired for Apo-YLOV at concentrations of 17.5 (red line), 35 (green line) and 70 µM (black line). Samples display characteristics of a self-associating species in monomer-dimer equilibrium with less than 3% of other contaminants. See text for details. (B) c(S)-Distributions derived from SV data acquired for Apo-YtvA at concentrations of 15 (red line), 30 (green line) and 60 µM (black line). At the lowest concentration the sample displays only one single species with an approximated molecular weight of 61 kDa representing dimeric Apo-YtvA. The slower sedimenting species visible at higher concentrations represents most likely a misfolded fraction of monomeric Apo-YtvA unable to dimerize.

Sedimentation velocity (SV) experiments performed for Apo-YLOV at concentrations of 17.5, 35 and 70 μM, displayed sedimentation profiles characteristic for a self-associating species (Figure 2A). At the lowest loading concentration the c(S) distribution appears as a broad and extensively tailed single peak with a weight-averaged apparent sedimentation coefficient (S_wapp_) of 1.94 S. The pronounced peak tailing already suggests the presence of two differently sized species with the smaller one dominating quantitatively. Due to the low S/N ratio and the correspondingly poor resolution the ratio is difficult to assess. Two individual peaks are observable with increasing concentration, the relative amount of the larger species is increasing and the S_wapp_ of the major peak shifts slightly towards higher values. Either is indicative for a self-associating system [40]. For the 70 µM sample, the best-fit weight-averaged frictional ratio (f/f0), which was determined iteratively from spreading of the sedimentation boundary, is 1.24 and the S_wapp_ of the smaller and the larger species is 1.58 S and 2.52 S, respectively. The apparent molecular weight (MW) approximated from the frictional ratio and the sedimentation coefficients is 11.4 kDa for the smaller and 22.8 kDa for the larger species. Given a calculated theoretical MW of 14.5 kDa for monomeric Apo-YLOV, this indicates a monomer-dimer equilibrium. Homogeneity amounted to more than 95% for the observed species and higher oligomers were not present in significant amounts. The approximated MWs of both species are significant lower than expected. This is caused by an underestimated frictional ratio, which is not unusual for self-associating systems, because spreading of the sedimentation boundary is affected not only by diffusion, but also by the reaction kinetics [40]. The examined concentration range, as well as the low size difference between the monomer and the dimer do not allow any precise assumption about the dissociation constant. Derived from the c(S) distribution of the 70 µM sample, 27.1% of Apo-YLOV is monomeric and 72.9% is associated in dimers, which corresponds to a binding constant (K_D_) of about 14 µM, but since the peaks are not completely baseline-separated, this K_D_ is only an estimate. Sedimentation equilibrium (SE) experiments for determination of a more exact binding constant yielded no meaningful results, most likely due to the instability of Apo-YLOV over the long time course needed for SE experiments.

In contrast to Apo-YLOV, SV experiments performed with Apo-YtvA showed no concentration dependent changes of the sedimentation behavior. A c(S) distribution derived from the lowest loading concentration of 15 µM shows a single peak with S_wapp_ of 3.6 S (Figure 2B, red line). The molecular weight approximated from that value and the best-fit f/f0 (1.45) is 61.1 kDa, which clearly points to a dimeric state of the protein. This finding indicates a K_D_ for self-dimerization on the order of at least 1 magnitude lower than the loading concentration [40]. Interestingly, at higher loading concentrations an additional small peak is observable at 2.1 S (Figure 2B, green and black lines). The MW of the corresponding species is 29.2 kDa, which points to a monomeric form of Apo-YtvA. Since its relative amount of about 7% is invariant with respect to the loading concentration, this species represents most likely an incorrectly folded fraction of Apo-YtvA not able to dimerize.

Even though our AUC data derived for Apo-YLOV shows that loss of the chromophore reduces the K_D_ of the LOV-LOV self-dimerization by at least 2 orders of magnitude, Apo-YtvA still exists almost exclusively as a dimer in the concentration range analysed here. This can be explained by an additional interaction between the STAS domains. As we showed previously, this interaction is weak (~350 µM) [28], but in combination with the weakened LOV-LOV interaction this results in a higher total affinity. The tighter binding between the Apo-YtvA monomers is therefore the result of a bivalent binding pattern, suggesting that the structure of the STAS domains and the intra-molecular STAS-STAS interactions were restored in the refolding process as was already indicated by our NMR data.

### Affinity of the YtvA-LOV domain for RF, FMN, and FAD

**Figure 3:**
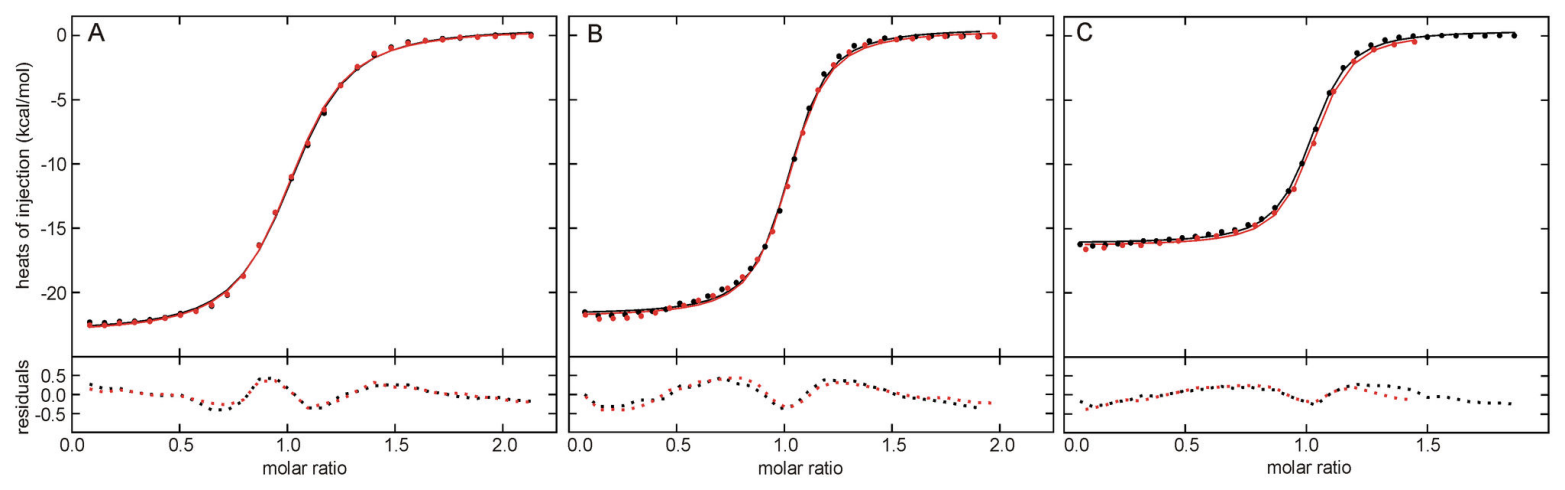
ITC binding isotherms from titrations of FMN, RF and FAD with Apo-YLOV. Titrations of 400 µM Apo-YLOV into 40 µM FMN (A), 40 µM RF (B) and 40 µM FAD (C) were performed twice and heats of reaction are represented in the upper diagrams by black and red dots, respectively. All titration curves were fitted for a single binding site.The individual binding isotherms (black and red lines) were obtained from a global fit of the combined data from experiments belonging to titrations with the same flavin. The corresponding residuals (rmsd) are displayed at the bottom. All calculated values are given in Table 2.

It has been demonstrated that some flavin binding photoreceptors including YtvA are able to incorporate natural as well as artificial or chemically modified flavins like roseoflavins in vivo and in vitro [10,14,15,33,37–39]. Since little is known about the affinity of LOV domains for the natural occurring flavins riboflavin, FMN and FAD, we analysed the thermodynamics of the interaction between these flavins and Apo-YLOV using Isothermal Titration Calorimetry (Figure 3).

The titrations were fitted for a single binding site and the fraction of the protein actively participating in binding amounted to at least 95% of the total protein. All calculated thermodynamic data are given in Table 2 and a thermogram derived from the control experiment to monitor dilution effects is given in Figure S4 in the supplementary material. Dilution of the protein produced only very small heat which was more than 1 order of magnitude lower than the heat produced by flavin binding. All interactions are driven enthalpically and display quite high binding constants in the submicromolar range. Unexpectedly, stronger binding is observed for RF and FAD than for FMN. Nevertheless, the thermodynamic binding profiles are quite similar for FMN and RF with respect to ∆H and ∆S indicating that the phosphate group of FMN does not provide an energetic contribution to the binding and, therefore, might not be necessary. In contrast, the binding mechanism of FAD seems to be different, because the interaction releases less heat and the decrease in entropy is significantly lower. Based on the crystal structure of the YtvA LOV domain [29], the latter can be explained by the fact that the flavin moiety fits well into the binding pocket while the adenosine part would move freely outside of the protein.

**Table 2:** Results of the ITC experiments performed with Apo-YLOV.

**ligand**	**binding active Apo-YLOV**	**∆H** (kcal / mol)	**T∆S** (kcal / mol)	**K_D_** (nM)	**∆G** (kcal / mol)
FMN	96.2%	-24.52 [-24.88; -24.16]	-16.14	715 [645; 791]	-8.38
RF	94.7%	-22.25 [-23.23; -22.46]	-13.45	351 [292; 397]	-8.80
FAD	98.3%	-16.57 [-16.86; -16.30]	-7.50	224 [192; 260]	-9.07

Values in brackets display the upper and lower limits within a 95% confidence interval.

### UV-Vis spectroscopy

The isoalloxazine ring and the ribityl side chain of all three flavins are most likely coordinated identically within the binding pocket, which is supported by the fact that all reconstituted YLOV variants were photochemically active with respect to the blue-light induced formation of the covalent flavin-cysteinyl-adduct and the thermally driven recovery of the ground state in the dark (see below). Light induced formation of the covalent adduct, the initial step within the photocycle of all LOV domains [41], is characterized by a decrease of typical flavin absorption at wavelengths larger than 410 nm and by a 60 nm hypsochromic shift of the 450 nm flavin absorption maximum after illumination. Subsequent analysis of corresponding UV-Vis spectra reveals that this applies to all reconstituted YLOV variants (see Figure S5 in the supplementary material).

### NMR of reconstituted LOV domains

**Figure 4:**
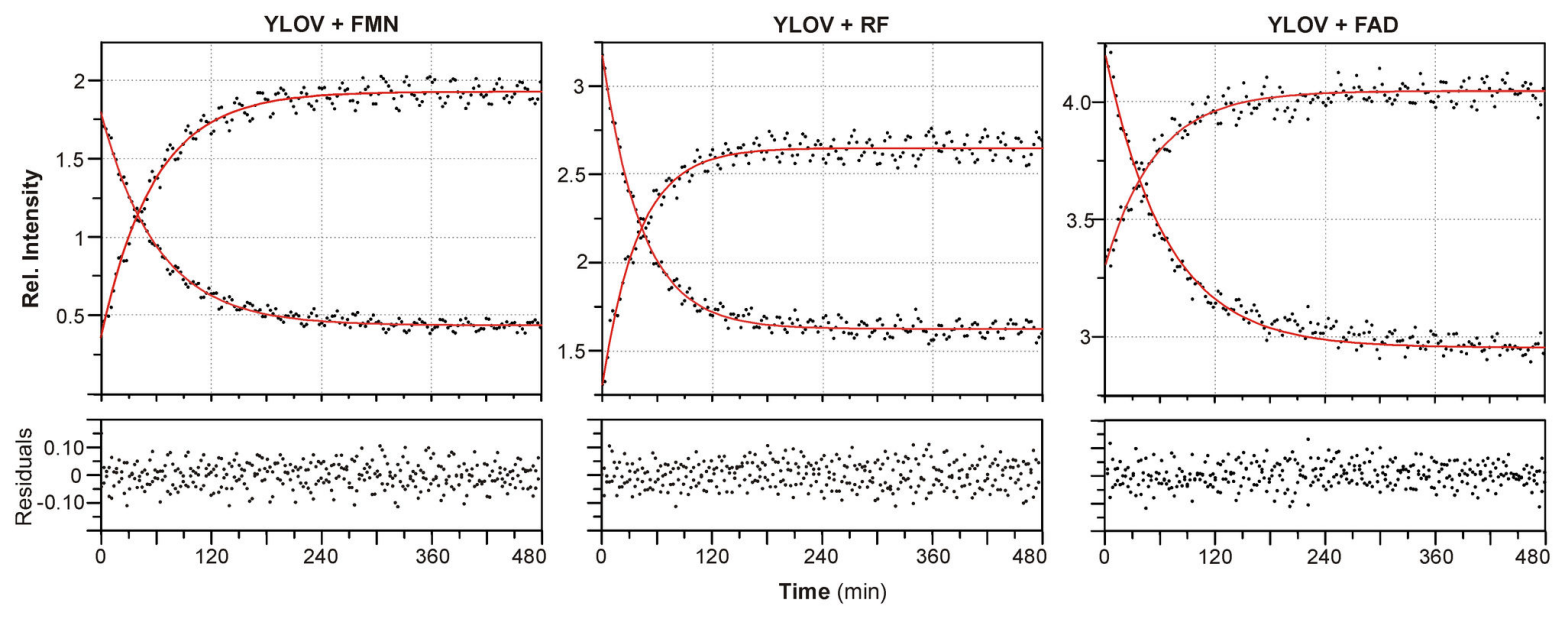
Ground state recovery of YLOV reconstituted with FMN, RF or FAD. Ground state recovery of YLOV either reconstituted with FMN, RF or FAD was monitored by tracking the intensity changes of a rising and a falling resonance signal within ^1^H-NMR spectra continuously recorded during the conversion process. Two representative ^1^H-NMR spectra including the monitored resonance signals are given in Figure S6 in the supplementary material. The intensities were plotted against time and fitted using a mono-exponential decay function (red line) with the calculated half-lives given in Table 3. The corresponding residuals (rmsd) are displayed at the bottom.

To test whether the exchange of the chromophore has an influence on the thermal recovery of the ground state, we determined the rate of recovery for all reconstituted proteins by tracking intensity changes of two distinct signals within pseudo-2D ^1^H NMR spectra (see Figure S6 for details). The complete list of lit state half-lives (T_1/2_) is given in Table 3. In E.coli-YtvA or E.coli-YLOV thermal recovery to the ground state occurrs within hours. We find very similar recovery times for FMN-YtvA and FMN-YLOV (Table 3) indicating a successful reconstitution procedure (Figure 4). Interestingly and in line with other results [33], the absence of the phosphate group in RF accelerates the photocycle by about 30% compared to FMN and FAD. This observation can be explained by an altered hydrogen-bonding pattern of residues surrounding the terminal hydroxyl group of RF. Since it has been suggested that recovery of the ground state is a base-catalysed reaction involving proton-transfer steps, modifications of the chromophore associated hydrogen-bonding network would indeed increase or decrease the recovery rate constant [42]. Additionally, electrostatic side-chain interactions between R^79^ and especially of R^63^ and the phosphate group (see Figure S7) might also contribute significantly to a change in recovery rates. R^63^ might stabilize the 3_10_-helix harbouring C^62^ and indirectly affect the stability of the covalent adduct between C^62^ and C4a of the chromophore. The latter interpretation is also supported by NMR data presented here.

**Table 3:** Lit state half lives of flavin-reconstituted YtvA and YLOV.

**protein**	**chromophore**	**T_1/2_** (min)
E.coli-YtvA	FMN	46.1 ± 1.7
FMN-YtvA	FMN	42.5 ± 0.1**^[a]^**
RF-YtvA	RF	21.8 ± 0.1**^[a]^**
E.coli-YLOV	FMN	42.4 ± 0.3
FMN-YLOV	FMN	41.3 ± 0.9
FAD-YLOV	FAD	42.4 ± 1.3
RF-YLOV	RF	28.6 ± 0.8

[a] values from UV-Vis spectroscopy following absorption at

450 nm during dark state recovery

**Figure 5:**
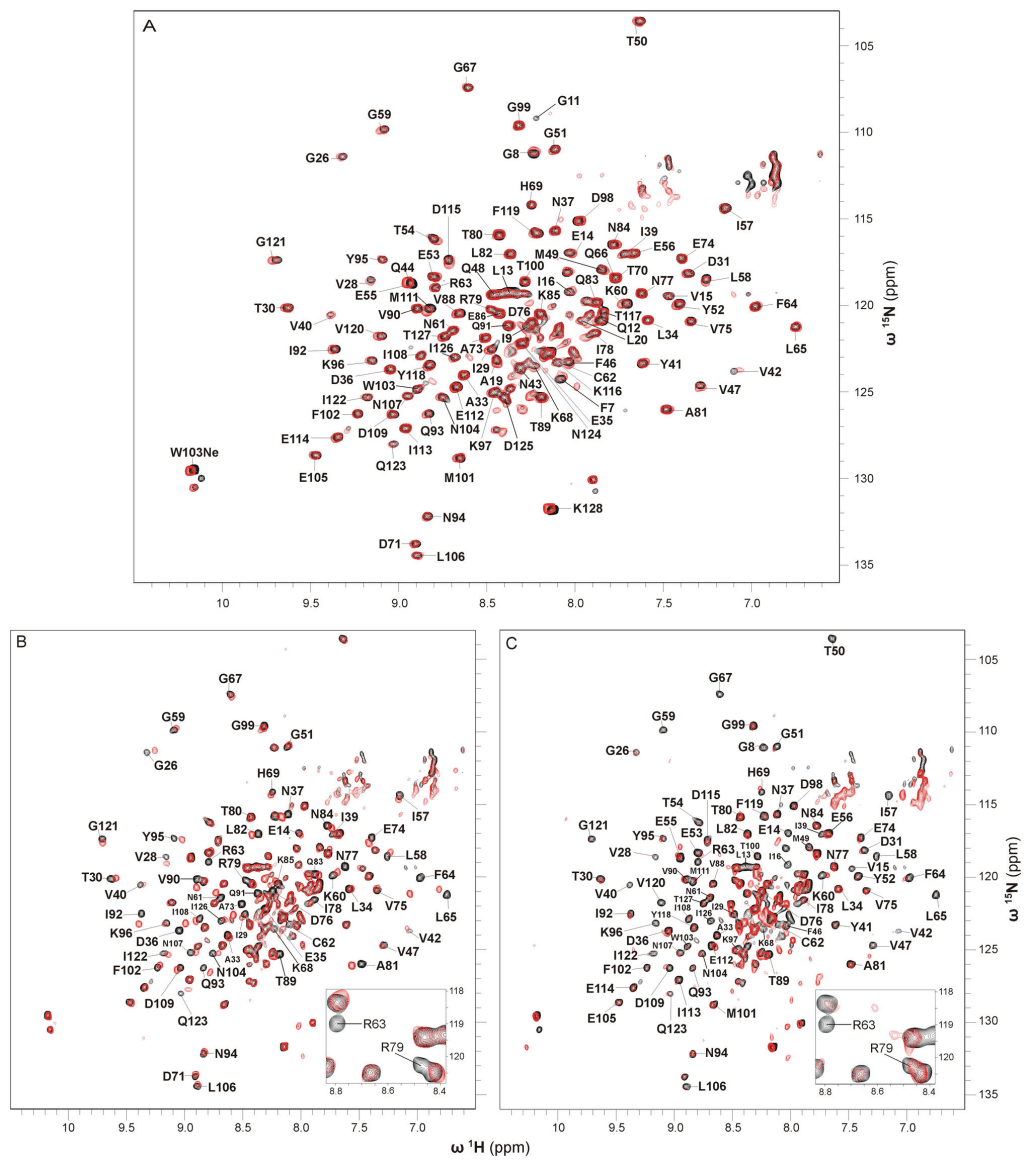
Superposition of ^1^H-^15^N-TROSY spectra of E.coli-YLOV and its reconstituted variants. (A) ^1^H-^15^N-TROSY spectra of E.coli-YLOV (black) and FMN-YLOV (red). All sequentially assigned resonances of E.coli-YLOV are labeled with residue type and sequence position. (B) ^1^H-^15^N-TROSY spectra of FMN-YLOV (black) and RF-YLOV (red). Sequentially assigned resonances of FMN-YLOV possessing different chemical shifts in RF-YLOV are labeled. Note that the signal from T^80^ is not shifted indicating that the chemical environment of this residue is the same in both proteins. (C) ^1^H-^15^N-TROSY spectra of FMN-YLOV (black) and FAD-YLOV (red). All both evaluable and sequentially assigned resonances of FMN-YLOV that are either changed in terms of intensity, shifted or unaffected in FAD-YLOV are labeled.The insets in A and B show a close up of the region of chemical shifts involving R^63^ and R^79^. All spectra were recorded from uniformly ^15^N-labeled samples.

**Figure 6:**
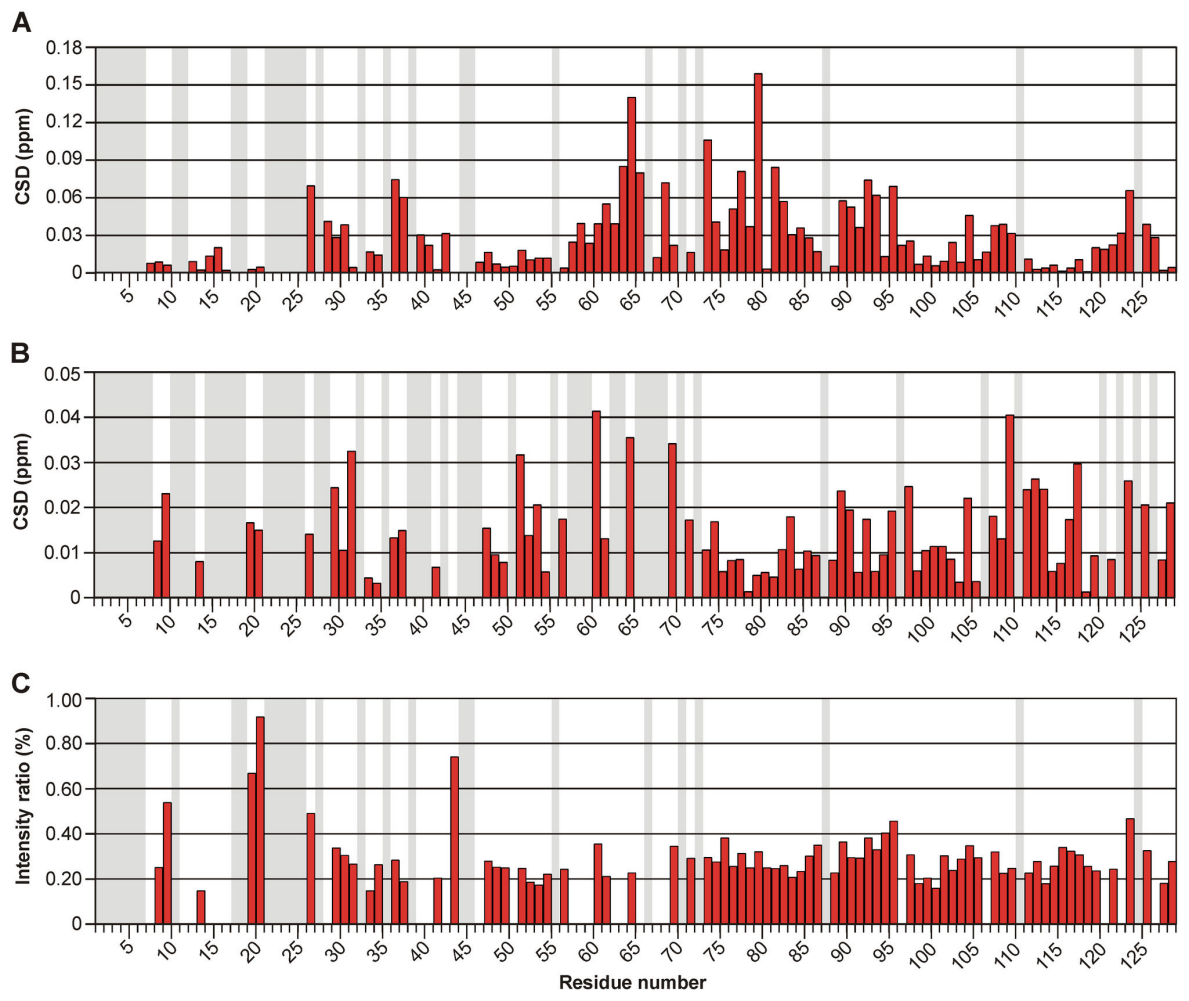
Chemical shift differences and crosspeak intensity ratios of YLOV reconstituted with RF or FAD. (A) Chemical shift differences (CSD) between ^1^H-^15^N-TROSY crosspeaks of FMN-YLOV and RF-YLOV. (B) CSD between ^1^H-^15^N-TROSY crosspeaks of FMN-YLOV and FAD-YLOV. (C) Intensity ratios of ^1^H-^15^N-TROSY crosspeaks of FMN-YLOV and FAD-YLOV. A gray background indicates residues that could not be assigned or evaluated. A complete list of all values is given in Table S1 in the supplementary material.

Besides providing evidence for the photochemical activity, NMR experiments additionally revealed that reconstituted apoproteins adopt the very same overall fold as the native proteins. In a superposition of ^1^H,^15^N-TROSY spectra from E.coli-YLOV and FMN-YLOV both proteins exhibit a nearly identical resonance pattern with respect to the intensities and positions of the individual amide resonances (Figure 5A). The ^1^H,^15^N-TROSY of RF-YLOV also shows a signal pattern very similar to FMN-YLOV. The absence of the phosphate group, however, leads to pronounced frequency shifts of the two arginines R^63^ and R^79^ (Figure 5B) and also changes the chemical shifts of some residues in the vicinity. Within the 3_10_-Helix forming highly conserved LOV consensus sequence (G(K)NCRFLQ) [2] where R^63^ is located, residues G^59^ to C^62^, F^64^ and L^65^ are affected. Except T^80^, chemical shifts of residues A^73^ to I^78^ and A^81^ to N^84^, all located in proximity to R^79^ inside the so called Fα-helix, also display significant changes. This also applies to residues located in the central beta-sheet (G^26^, V^28^ to T^30^, I^39^, V^40^, V^42^ T^89^ to Q^93^, Y^95^, F^102^, N^104^, N^107^ to D^109^ and G^121^ to Q^123^) as well as D^36^ and N^37^ located in the loop between strands Aβ and Bβ. Based on the crystal structure of the YtvA LOV domain [29], in which the carboxyl group of D^36^ forms polar contacts to the guanidinium group of R^63^, this suggests that these contacts are somehow altered if the terminal phosphate group is not present. Exchange of FMN by RF also affects the chemical environment of further residues located in loops (I^57^, L^58^, K^68^, H^69^, K^96^, K^97^, D^125^ and I^126^). For better visualisation we performed a minimum chemical shift analysis and plotted the magnitude of the chemical shift differences (CSD) against the residue number (Figure 6A). Additionally, a complete list of all assigned chemical shifts and CSD is given in Table S1 in the supplementary material.

A comparison of ^1^H,^15^N-TROSYs of FMN-YtvA and RF-YtvA showed that absence of the phosphate group affects the same residues which displayed pronounced chemical shift differences between FMN-LOV and RF-YLOV, but does not have an effect on the STAS domain (Figure S8). Based on the observed chemical shift differences, it is not possible to predict the actual structural changes, but considering that the chemical environment of most residues is virtually unchanged and that RF-YtvA is photoactive, it can be assumed that the protein exhibits the very same global structure as the wild-type, except only very small local differences in chromophore-surrounding regions (i.e. orientation of side chains and interatomic distances).

In contrast, FAD has a considerably stronger influence on the LOV structure. A comparison between the ^1^H,^15^N-TROSYs from FMN-YLOV and FAD-YLOV shows that about 30 cross-peaks are extremely weak or have completely disappeared and that most of the remaining signals are notably diminished (Figure 5C). Additionally, some of the latter are slightly shifted. A plot of all evaluable CSD as well as a plot of the observed crosspeak intensity ratios against the residue numbers is given in Figure 6B and 6C, respectively. A complete list of all assigned chemical shifts, the CSD and the intensity ratios is given in Table S1 in the supplementary material. The pronounced intensity loss is mainly visible at resonances of amino acids within the central beta-sheet (V^28^, I^39^, to V^42^, L^106^, I^113^,V^120^and I^122^), and of those from the loop connecting strands Aβ and Bβ (A^33^ and N^37^), the conserved 3_10_-helix including its sequentially adjacent loops (I^57^ to G^59^, C^62^, R^63^, L^65^, G^67^ and K^68^), the α-helix Cα (F^46^, T^50^) including its adjacent loop (Y^52^, E^53^) and residues within further loops (K^96^, D^98^ to T^100^, I^126^ and T^127^). The most likely explanation is the presence of intermediate exchange with respect to the NMR timescale. This also applies to residues F^7^ and G^11^ to I^16^ of the N-terminus. Only 10 residues distributed throughout the whole sequence (D^31^, G^51^, K^60^, F^64^, H^69^, K^97^, D^109^, E^112^, T^117^ and Q^123^) show pronounced chemical shift differences (CSD > 0.025 ppm). Beside the central beta-sheet, FAD exchange mainly affects structural elements opposite to the dimer interface and within the Ncap-sequence. So it seems likely that the adenosine moiety dynamically interacts with residues of these regions. Such additional weak interactions could also be an explanation for the slightly higher affinity of FAD.

Since the residues coordinating the ribityl chain and the phosphate group of FMN are highly conserved in phototropin and phototropin-like LOV domains (aa N^61^, R^63^, Q^66^ and R^79^ in YtvA) [2,29,43], binding specificity of YLOV for FMN, RF and FAD presented in this work should be comparable to other phot-like LOV domains. This is also supported by the fact, that the backbones of the LOV structures given in Table 1 are extremely similar and do not provide any explanations whether certain domains could have different flavin specificities. The average Cα-RMSD calculated from a structural alignment of the LOV domains in Table 1 to the LOV domain of YtvA (PDB-ID: 2PR5 [29]) using the CEAlign algorithm [44] implemented in Pymol v1.5.0.4 [45] is 1.67±0.35 Å. An exception could be LOV domains possessing an extended loop between the conserved consensus sequence and its subsequent α-helix (i.e. the LOV domain of Vivid (PDB-Entry: 2PD7) [21] and WC-1 [20]), because an additional polar interaction between the pyrophosphate of FAD and the side-chain of a lysine within that loop may enhance its affinity for FAD.

### Use of the YtvA LOV domain as a molecular probe for free flavins in cell lysates

Since phot-like LOV domains bind RF, FMN and FAD with comparable affinities, flavin composition of heterologously overexpressed LOV proteins should reflect the relative amount of available cytosolic flavins during the expression phase. The cytosolic flavin composition, however, seems to vary considerably depending on the chosen expression host (i.e. insect cells, fungi, *E. coli*), the culturing conditions (i.e. media, temperature, duration) and the sequence of the protein [3,11–13,15,16,20]. For that reason, chromophores found in a recombinantly expressed LOV protein are not necessarily the physiological chromophores. Testing the physiological chromophore composition, however, is not trivial, since purification of the desired endogenous protein from its source organism is required. Knowledge of a receptor’s affinity for the natural occurring flavins combined with information on the absolute cytosolic concentrations of these flavins in the source cell it should allow an estimate of physiological chromophore composition in vivo. Unfortunately, absolute cytosolic flavin concentrations are also not easily obtained [46,47]. In contrast, use of affinity-tagged Apo-YLOV could provide a simple method for an indirect determination of the chromophore composition and was tested here for YtvA in wild-type *Bacillus subtilis* cells.

**Figure 7:**
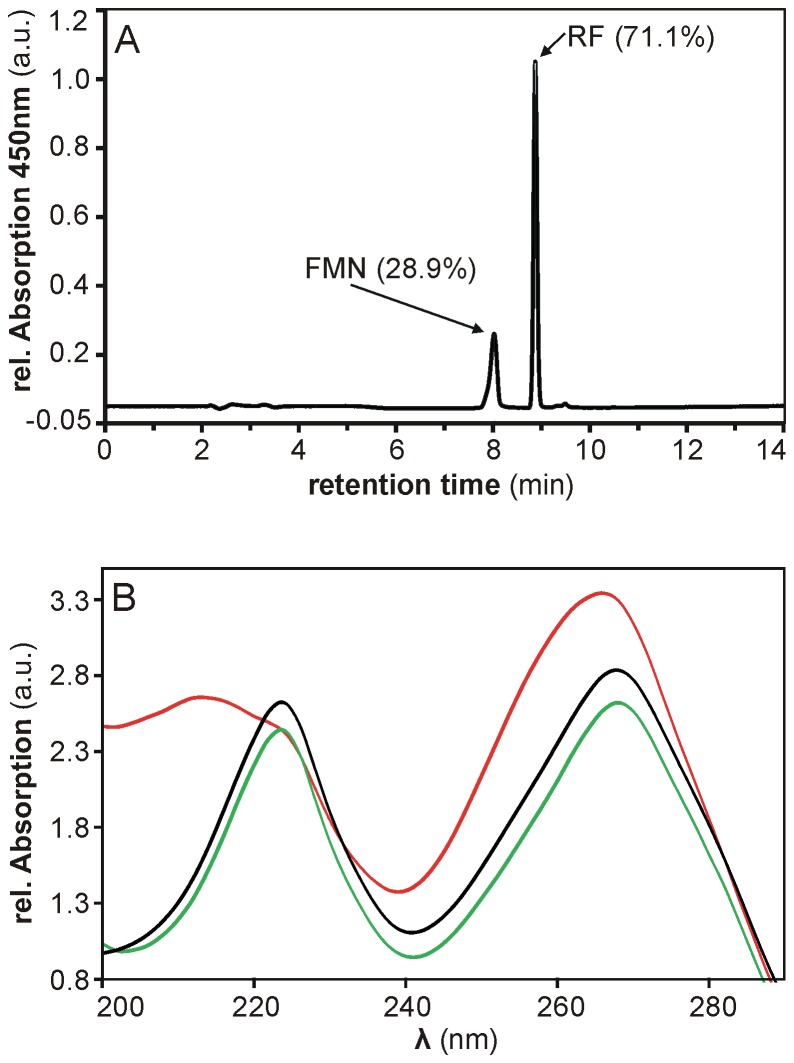
HPLC-based analysis of flavins isolated from *B. subtilis* cell lysate. (A) By means of reverse-phase HPLC, flavins isolated from *B. subtilis* cell lysate using His-tagged Apo-YLOV as a molecular probe were identified as RF and FMN. The corresponding peaks are labeled by type of flavin and its relative amount. (B) Comparison of UV-Vis spectra measured at the respective peak maxima of FMN from *B. subtilis* (black line) and FMN (green line) and FAD (red line) from reference samples indicates that the faster eluting flavin species isolated from *B. subtilis* is indeed FMN.

We used the lysate derived from *B. subtilis* cells in the stationary phase. In a HPLC-based analysis of the chromophores released from lysate-incubated Apo-YLOV two individual flavin species with retention times (t_R_) of 8.88 min and 8.04±0.05 min were detected (Figure 7A). Based on calibrations with RF (t_R_ = 8.88 min), FMN (t_R_ = 8.17±0.01 min) and FAD (t_R_ = 7.58±0.01 min), one signal clearly corresponds to RF, whereas the retention time of the second peak points to FMN but slightly shifted towards FAD. The UV-Vis spectrum recorded at the peak maximum, however, uniquely corresponds to that from the FMN reference suggesting that it is indeed FMN (Figure 7B). This is particularly evident in the spectral range below 220 nm where only FAD exhibits an additional absorption maximum at 212 nm caused by the adenosine moiety [48].

With respect to the total flavin content, the sample contained about 71% RF and 29% FMN but no detectable amount of FAD suggesting that endogenous YtvA should exhibit a comparable flavin composition. The method does not allow any conclusion about the absolute cytosolic flavin concentration but taking the binding constants into account FMN and RF appear to be present in equimolar concentrations. This result together with the fact that the cells did not contain sufficiently free FAD is somewhat surprising because RF is usually quickly converted into FMN and FAD [49]. An explanation could be the physiological constitution of the stationary-phase cells suffering from either an impaired FAD biosynthesis or a generally lower flavin content. Although Apo-YLOV binds FAD with three times the affinity of FMN, the protein likewise contained virtually no FAD upon heterologous expression in *E. coli* suggesting that the FAD pool is rapidly depleted at the beginning of the expression phase while all initially present and continuously synthesized FMN and also a little RF is captured by continuously increasing amounts of apoprotein. This effect could be directly associated with the expression rate and would explain the varying flavin compositions found in heterologously expressed LOV proteins.

Moreover, the protein has potential to be used as a flavin specific molecular probe for the determination of flavin ratios in soluble fractions from cell lysates. This would, however, require a further validation of the method, testing whether the affinity-tagged protein can reliably reproduce the flavin composition of standard mixtures and is not influenced by the composition of the flavin pool.

## Conclusions

We have established a robust protocol for the efficient preparation of the apo form of the bacterial phototropin-like photoreceptor YtvA and its isolated LOV domain that allows complete conversion of holoproteins into soluble apoproteins. Both Apo-YtvA and Apo-YLOV can be fully reconstituted with RF, FMN and FAD under physiological conditions and we show here that all reconstituted variants are photochemically active and adopt the same structure as the respective holoproteins prepared under non-denaturating conditions. The results clearly show that the chromophore stabilizes the holoprotein since its absence alters the structure and the conformational dynamics of the whole LOV domain leading to a significant weakening but not to a loss of the LOV-LOV interaction. Beyond that the results confirmed our assumption [26] about the presence of intra-molecular interactions between the STAS domains resulting in a dimerization of YtvA.

By means of ITC experiments, we have studied the thermodynamics of the interaction of Apo-YLOV with RF, FMN and FAD and found that the YtvA-LOV domain binds all tested flavins with comparable affinities in the upper nanomolar range which means that this domain does not prefer FMN and that the terminal phosphate of FMN might not be necessary for binding. Comparison of ^1^H,^15^N-TROSY spectra of the reconstituted proteins indicated that variants containing RF or FMN adopt virtually the same global structure with only very small differences in chromophore surrounding regions. Absence of the phosphate group most likely changes the hydrogen bonding network within the binding pocket which explains the accelerated dark-state recovery of RF-YtvA and RF-YLOV. In contrast, YLOV associated with FAD has an enhanced conformational flexibility most likely caused by transient interactions of the adenine moiety with residues on the protein surface.

All tested flavins exert a stabilizing effect on the conformation of the LOV domain. On the basis of sedimentation velocity experiments performed with Apo-YtvA and Apo-YLOV, we demonstrate that the loss of the chromophore reduces the binding constant for LOV-LOV self-dimerization by at least two orders of magnitude and we confirm our previous assumption that dimerization of YtvA is indeed forced not only by intermolecular LOV-LOV contacts but also by interactions between the STAS domains.

Finally we demonstrate the application of Apo-YLOV as a molecular probe for free flavins in cell lysate from *Bacillus subtilis* and found that endogenous YtvA is associated with 71% RF and 29% FMN.

## Materials and Methods

### Buffer

30 mM Tris-HCl pH = 7.9; 300 mM NaCl; 1 mM MgSO_4_; 5 mM imidazole; 10% glycerol; Complete protease inhibitor mix (Roche)
20 mM Na-Phosphate pH = 7.4; 50 mM NaCl; 1 mM DTT
20 mM Tris-HCl pH = 7.5; 150 mM NaCl; 8 M urea
20 mM Tris-HCl pH = 7.5; 150 mM NaCl; 6.5 M urea
20 mM Tris-HCl pH = 7.5; 150 mM NaCl; 1mM DTT
20 mM Tris-HCl pH = 7.5; 150 mM NaCl; 400 mM imidazole
20 mM Tris-HCl pH = 7.5; 150 mM NaCl; 500µM TCEP
20 mM K-Phosphate pH = 6.5, 50 mM NaCl, 5 mM DTT; 0.1% azide

### Cloning

Construction of the expression plasmids for full length YtvA (aa 1 – 261) and for the LOV domain of YtvA (YLOV, aa 1 – 128) was performed as described earlier [27]. For cloning of YLOV the following primers were used:

Forward tobacco etch virus (TEV): 5’- gacgacgacaagatggaaaacctgtatttccag-3’
Forward YLOV: 5’- aacctgtatttccagggagctagttttcaatcatttgggatac-3’
Reverse YLOV: 5’- gaggagaagcccggtttacttggtgatatcattctga-3’

As described earlier [27], a TEV cleavage site was introduced, leaving an additional Gly at the N-terminus after proteolysis. In YtvA the wild-type Met^1^ was replaced by this glycine. All clones were checked by bidirectional DNA sequence analysis (Invitek, Berlin, Germany).

### Protein expression & purification of holoproteins

Expression and purification of unlabeled and ^2^H^15^N-labeled YtvA was done following the protocols of Jurk et al. [27,28]. Expression of ^15^N-labeled YLOV was done similar to the method previously described for the expression of the ^15^N-labeled YtvA-STAS domain, except the expression temperature, which was kept at 25 °C. For details see Dorn et al. [50]. Recombinant expression of unlabeled YLOV was also carried out in a high cell density fermentation system (FedBatch Pro, DASGIP, Germany) using T7-Express Rosetta2 cells as expression host (New England Biolabs GmbH, Frankfurt a.M., Germany). Unlabeled YLOV was expressed in TB medium supplemented with 2% glucose and the appropriate antibiotics. Two pre-cultures of 400 ml TB (supplemented as described above) were inoculated from an overnight culture to an OD_600_ of 0.1 and cells were grown at 37 °C to a final OD_600_ of 2.0 - 2.5. Cells were harvested by mild centrifugation (1000 x g, 10 min) at room temperature and the pellet was resuspended in 50 ml TB medium. This amount was used for the inoculation of a 1000 ml bioreactor. During the batch phase the temperature was kept constantly at 37 °C. The pH of the media was kept above 7 during the whole fermentation process by stepwise addition of 1 M NaOH. After reaching an OD_600_ between 14 and 18 the bioreactor was cooled to 25 °C and 60 ml expression feed (15 g glucose, 3.5 g NH_4_Cl in TB media) was pumped into the bioreactor with a flow rate of 6 ml/h. The induction was started 30 min afterwards by the addition of IPTG to a final concentration of 1 mM. 1 h after complete addition of the expression feed cells were harvested by centrifugation in a pre-chilled rotor at 4 °C and either stored at -80 °C or, after resuspension in buffer A, directly lysed with an EmulsiFlex-C3 High-Pressure Homogenizer (Avestin Inc., Ottawa, Canada).

Unlabeled and ^15^N-labeled YLOV fusion proteins were purified by affinity chromatography on a Poros 20mc column (Workstation Vision, Applied Biosystems) using recommended standard buffers.

TEV cleavage and further purification of YLOV was performed as described earlier [50] except that buffer B was used in the size exclusion chromatography. Riboflavin incorporated in heterologously expressed YtvA and YLOV not used for apoprotein production was exchanged against FMN. For details please see Protocol S1 in the supplementary material.

### Preparation of apoproteins

His-tagged fusion protein was kept in the dark for at least 12 h at room temperature. To release the chromophore, the protein solution was mixed with an appropriate amount of buffer C (final urea concentration of 6.5 M) and subsequently incubated on a rotary shaker for 3 h. The solution was loaded on a pre-equilibrated 5ml His-Trap column (HisTrap HP; Pharmacia Biotech) and the chromophore was rinsed out with 10 column volumes (CV) of buffer D. Refolding of the protein was achieved by washing with 10 CV of buffer E at a flow rate of 0.5 ml min^-1^. The apoprotein was eluted using buffer F. Imidazole was removed by dialysis in a 1000-fold excess of buffer E. TEV cleavage and further purification were performed as described above for the correspondent holoproteins. Complete removal of the chromophore was confirmed by the absence of the typical flavin absorption within UV-Vis spectra.

### Reconstitution of apoproteins

Reconstitution of apoproteins with the desired flavins was done either on column directly after refolding or in solution during ITC experiments (see ITC section). On-column reconstitution was achieved by incubation with 5 CV of buffer E containing 100 µM of the desired flavin at a flow rate of 0.5 ml min^-1^. Prior to elution excess chromophore was washed away with 10 CV of buffer E. TEV cleavage and further purification were performed as described above for the correspondent holoproteins.

### Analysis of *Bacillus subtilis* flavins

Four shaking flasks with 400 ml media (4 g/l meat extract; Roth) were inoculated from a glycerol stock of *Bacillus subtilis* wild-type strain DSM402 and incubated at 30 °C and 60 rpm in the dark until measurements of OD_600_ indicated entry into stationary phase (final OD_600_ was 2.5 ml^-1^). Cells were harvested by centrifugation, the resulting pellet (8.7 g bio wet mass) washed twice with 25 ml 150 mM NaCl solution and then resuspended in 40 ml of buffer A. Cells were lysed with an EmulsiFlex-C3 High-Pressure Homogenizer (Avestin Inc., Ottawa, Canada) and the lysate was clarified by centrifugation. 20 mg of His-tagged YLOV apoprotein were loaded on a pre-equilibrated 5 ml His-Trap column (HisTrap HP; Pharmacia Biotech) and incubated with the lysate by pumping the latter twice over the column with a flow rate of 1 ml min^-1^. After washing with 10 CV of buffer E YLOV was eluted with 3 CV of buffer F. Imidazole was removed by buffer exchange (buffer E) during 3 concentration / dilution steps. The solution was then concentrated to 1 ml and stored in the dark at 4 °C for 48 h. The bound chromophores were released by heat denaturation (95 °C for 5 min in the dark). Afterwards the solution was thoroughly mixed with a vortex and clarified by centrifugation at 4 °C. To get rid of dissolved proteins the supernatant was filtrated using a centrifugal filter device (Vivaspin; 2 kDa MWCO) and the filtrate was analysed by HPLC (HP-1100 series; Hewlett Packard) using a Nucleodur C18 pyramid column (250 mm length; 5 mm diameter; Macherey-Nagel). The chromophores were separated with a linear water / acetonitrile gradient (5-95%; 1 ml min^-1^ flow rate). Detection was carried out at 280 nm and 450 nm. Additionally, a complete UV-Vis spectrum (200-800 nm) was recorded at every data point of the chromatogram. Retention times of RF, FMN and FAD were determined separately using stock solutions (10 µg ml^-1^ in H_2_O) of the single flavins. All chromatographic runs were repeated three times and the retention times of corresponding signals were averaged.

### Sample preparation

Riboflavin present in commercially available FMN (up to 15%) was removed quantitatively by anion exchange chromatography. 200 mg FMN from Sigma-Aldrich were dissolved in 20 ml H_2_O and loaded on a self-packed 10 ml Q-Sepharose column (Q-Sepharose FF; GE-Healthcare). After washing with 10 CV H_2_O the bound FMN was eluted with 4 CV of 150 mM NaCl solution. The remaining riboflavin content was checked by HPLC to be less than 3%.

Proteins used for Isothermal Titration Calorimetry were dialysed for 24 h at 8 °C against 1000-fold excess of buffer G. FMN, FAD (Sigma-Aldrich), and RF (Roth) were not dialysed because of their low molecular weight. Desired concentrations were achieved by dilution with the appropriate dialysis buffer. Concentrations of the flavins were calculated from the absorption at 450 nm using a molar extinction coefficient of ε = 12500 M^-1^ cm^-1^ for RF and FMN and ε = 11300 M^-1^ cm^-1^ for FAD. Concentration of Apo-YLOV was calculated from the absorption at 280 nm using a calculated molar extinction coefficient (ProtParam) of ε = 11460 M^-1^ cm^-1^. NMR experiments were done in buffer H for YtvA and buffer B for YLOV, respectively. Additionally, all NMR samples contained 5-10% D_2_O. Prior to NMR spectroscopic investigation of flavin reconstituted YLOV generated in ITC experiments samples of both titrations were pooled and an excess of the corresponding flavin was added to a final concentration of 70 µM. After 30 min incubation at room temperature the solution was dialysed for 24 h against 5 l of buffer E at 8 °C and then concentrated to 500 µl. ^15^N-labeled Apo-YLOV used in ITC control experiments (see ITC section) was treated similarly, but no flavins were added. Proteins used for analytical ultracentrifugation were dialysed for 24 h at 8 °C against a 1000-fold excess of buffer H in case of Apo-YtvA and buffer G in case of Apo-YLOV, respectively. Concentrations were calculated from the absorption at 280 nm using a calculated molar extinction coefficient (ProtParam) of ε = 16055 M^-1^ cm^-1^ for Apo-YtvA and the above mentioned value for Apo-YLOV.

### Analytical Ultracentrifugation

Sedimentation velocity experiments were performed with a Beckman Optima XL-I analytical ultracentrifuge equipped with absorption and interference optics. A four-hole rotor and two-sector titan or Epon cells were used. Experiments were performed at a rotor speed of 45000 rpm at 20 °C for Apo-YtvA and 25 °C for Apo-YLOV, respectively. Data were acquired as absorbance scans with a time interval of 5 min for Apo-YtvA or 8 min for Apo-YLOV. Absorbance was measured at 280 nm, with radial increments of 0.003 cm. Loading Volume was 400 µl of 15, 30, and 60 µM Apo-YtvA and 17.5, 35, and 70 µM Apo-YLOV, respectively. Data evaluation was done using the software Sedfit v13.2 [51,52]. Buffer density and viscosity as well as partial specific volume of the proteins were calculated using Sednterp v1.09 [53].

### Isothermal Titration Calorimetry

Isothermal Titration Calorimetry experiments were performed with a VP-ITC microcalorimeter (Microcal) at 25 °C. All titrations were performed twice. During titrations the injection unit was shielded from incident light to prevent photo conversion of reconstituted proteins. The protein solution (400 µM ^15^N-labeled Apo-YLOV) was titrated into the sample cell containing either 40 µM FMN, 40 µM FAD or 40 µM riboflavin. The volume of each injection was either 7 or 10 µl except the first one where only 2 µl were injected. Spacing time between each injection was 5 min. Control experiments to monitor dilution effects were performed by titration of 400 µM Apo-YLOV into the sample cell containing only buffer. Data processing was performed using the Microcal Origin software. Baseline correction and peak integration were done manually. The association constant (K) and the enthalpy change (∆H) of the interactions were obtained from fitting of the experimental titration curve for a single binding site using the software Sedphat v10.54e [54]. The enthalpy, the association constant and the correction factor for the active protein concentration were kept variable during the fitting procedure. Finally, datasets belonging to titrations with the same flavin were fitted globally and a Monte Carlo based 95% confidence interval analysis of the resulting values of ∆H and K was performed using a function of Sedphat which is based on the method described by Kemmer et al. [55] and Broecker et al. [56]. Gibbs free energy (∆G) and the entropy change (∆S) were calculated using the equation ∆G = -RT ln(K) and ∆S = (∆H -∆G)/T, respectively.

### NMR-Experiments

NMR experiments were performed at 300 K on a Bruker Avance 600 MHz spectrometer equipped with a 5 mm triple resonance PFG (z-axis) cryo probe head. All spectra were processed using Topspin 2.1 software. Heteronuclear NMR spectroscopic investigation of YtvA and YLOV was achieved by recording 2D ^1^H-^15^N-TROSY experiments [57,58]. Photochemical activity of the flavin-reconstituted proteins was checked by measuring the intensity change of two characteristic proton signals within 1D ^1^H-spectra during the dark state recovery (see Figure S6). This was achieved by time-incremented recording a series of 1D ^1^H-spectra within a pseudo-2D spectrum, in which the direct dimension represents the proton signals and the indirect dimension the time. 256 points with time increments of 168 seconds were recorded in the indirect dimension. The intensity values of the two characteristic ^1^H-signals were extracted from the pseudo-2D spectrum with Topspin and plotted against time with the Origin software. The half-life of the photoactive state was calculated by averaging the corresponding values obtained from the single exponential fits of both relaxation curves. For photo-conversion and maintenance of the photostationary state, the previously described fiber-coupled light source was used [50]. Calculation of chemical shift differences (CSD) and crosspeak intensity ratios from 2D heteronuclear NMR spectra was done using CCPNMR analysis v.2.2 software [59]. ^15^N chemical shifts were scaled by a factor of 0.1 for CSD calculation. 

## Supporting Information

Figure S1 **HPLC-based chromophore analysis of YLOV.** (A) Chromatogram of the flavins released from heterologously expressed YLOV proved the incorporation of 89% FMN and 11% riboflavin (RF) during overexpression in *E. coli*. (B) After exchange of RF by FMN under native conditions (see text), heterologously expressed YLOV contained no detectable amount of RF. HPLC was performed using a Shimadzu LC-6A chromatography station equipped with a C18-Pyramid column (Nucleodur; 5 mm diameter; 100 mm length). Elution was done with a linear acetonitrile gradient (0-80% in H_2_O) and detection was carried out at 280 nm and 447 nm.
(TIF)

Figure S2 **Absorption spectrum of YLOV and Apo-YLOV.** Spectra were derived from 30 µM heterologously expressed dark state YLOV before (black line) and after deflavination (red line). Absence of the typical flavin absorbance between 310 nm and 510 nm indicates complete chromophore removal. The spectra were measured using a DU-520 spectrophotometer (Beckman-Coulter).
(TIF)

Figure S3 **Superposition of ^1^H-^15^N-TROSY spectra of native and apo form of YtvA.** ^1^H-^15^N-TROSY spectra of native and deflavinated YtvA are shown in black and red, respectively. Both samples were uniformly ^2^H-^15^N-labeled. Sequentially assigned amide resonances of YtvA outside the central region of the spectrum are labeled with residue type and sequence position (SC: resonances most likely belonging to side chains). See main text for details.
(TIF)

Figure S4 **ITC thermogram of the titration of Apo-YLOV into buffer.** The upper panel displays the ITC thermogram derived from the titration of 400 µM Apo-YLOV into buffer. The heat generated by each injection is shown below. To illustrate the negligible dilution effects, the vertical axes were scaled to the same range used for evaluation of titrations performed with flavins. The titration experiment was performed at 25°C using a Microcal VP-ITC microcalorimeter.
(TIF)

Figure S5 **UV-Vis spectra of unexposed and illuminated YLOV reconstituted with flavins.** The spectra were obtained from YLOV reconstituted with FMN, RF or FAD during ITC experiments. Spectra of unexposed and illuminated proteins are shown in black and red, respectively. Absence of the typical chromophore absorbance pattern at λ > 410 nm after illumination with blue-light indicates formation of the covalent adduct between carbon C4a of the isoalloxazine ring and the sulfur within the side chain of cysteine C^62^ proving that all reconstituted variants were photochemically active. Photo conversion and accumulation of the photoactivated state were achieved by illuminating the samples contained in a 200 µl pipette tip for 30 s with a high power LED (H6-RGB-9; λ_max_ = 460 nm; Φ = 430 mW; Roithner Lasertechnik; Austria). The spectra were measured using a DU-520 spectrophotometer (Beckman-Coulter).
(TIF)

Figure S6 **Superimposed ^1^H-NMR spectra of dark-state and illuminated YLOV.** The ^1^H-spectra of dark-state (black) and illuminated YLOV (red) differ significantly indicating light-induced structural rearrangements. The signals enlarged in the insets were used to monitor the dark-state recovery kinetics by tracking its intensity changes during conversion of photo activated YLOV to the ground state. The intensity of the signal at -0.475 ppm (B) is maximum in the ground state going to zero after illumination whereas the signal at 6.46 ppm (A) displays opposing characteristics. Corresponding signals are also present in ^1^H-spectra of YtvA and YLOV variants containing RF or FAD. The signal at -0.475 ppm belongs to a methyl group of I108. The origin of the other signal (B) is unknown. To obtain the lit-state spectrum, sample was illuminated using our previously described fiber coupled light source [50]. Spectra were acquired at 27°C on a 600 MHz NMR spectrometer (Bruker Avance III) equipped with a 5 mm triple resonance PFG (z-axis) cryo probe head.
(TIF)

Figure S7 **Polar interactions between FMN and arginines R^63^ and R^79^ of the YtvA LOV domain.** Close-up of the YtvA LOV domain structure (PDB-ID: 2PR5 [29]) showing polar interactions (green dotted lines) between the phosphate group of FMN and the side chains of arginines R^63^ and R^79^. Heteroatoms are color-coded: nitrogen (blue), oxygen (red), phosphor (orange).
(TIF)

Figure S8 **Superimposed ^1^H-^15^N-TROSY spectra of YtvA reconstituted with FMN and RF.** The ^1^H-^15^N-TROSY spectrum of YtvA reconstituted with FMN and RF is colored in magenta and green, respectively. Both samples were uniformly ^2^H-^15^N-labeled. Resonances belonging to amino acids significantly or slightly affected by exchange of FMN against RF are labeled with residue type and sequence position (SC: Unassigned amide resonances most-likely belonging to side-chains). See main text for details. Spectra were acquired at 27°C on a 600 MHz NMR spectrometer (Bruker Avance III) equipped with a 5 mm triple resonance PFG (z-axis) cryo probe head.
(TIF)

Protocol S1 **Exchange of RF by FMN in YtvA and YLOV under native conditions.**
(DOCX)

Table S1 **Chemical shifts of YLOV reconstituted with FMN, RF and FAD.**
(PDF)
