# Supplementary figures and images for: LOV Takes a Pick: Thermodynamic and Structural Aspects of the Flavin-LOV-Interaction of the Blue-Light Sensitive Photoreceptor YtvA from Bacillus subtilis
Source: PLoS One. 2013 Nov 21;8(11):e81268. doi: 10.1371/journal.pone.0081268 (PMC3836802; doi:10.1371/journal.pone.0081268)

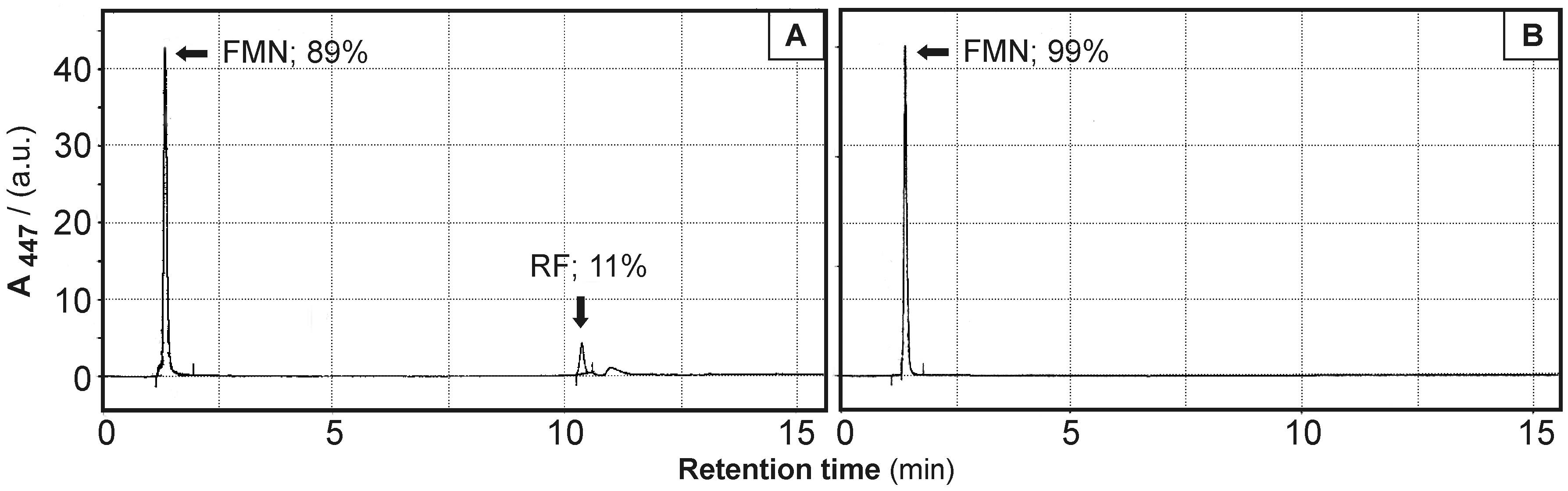

Supplement: Figure S1 — HPLC-based chromophore analysis of YLOV. (A) Chromatogram of the flavins released from heterologously expressed YLOV proved the incorporation of 89% FMN and 11% riboflavin (RF) during overexpression in E. coli. (B) After exchange of RF by FMN under native conditions (see text), heterologously expressed YLOV contained no detectable amount of RF. HPLC was performed using a Shimadzu LC-6A chromatography station equipped with a C18-Pyramid column (Nucleodur; 5 mm diameter; 100 mm length). Elution was done with a linear acetonitrile gradient (0-80% in H2O) and detection was carried out at 280 nm and 447 nm. (TIF) [file pone.0081268.s001.tif]

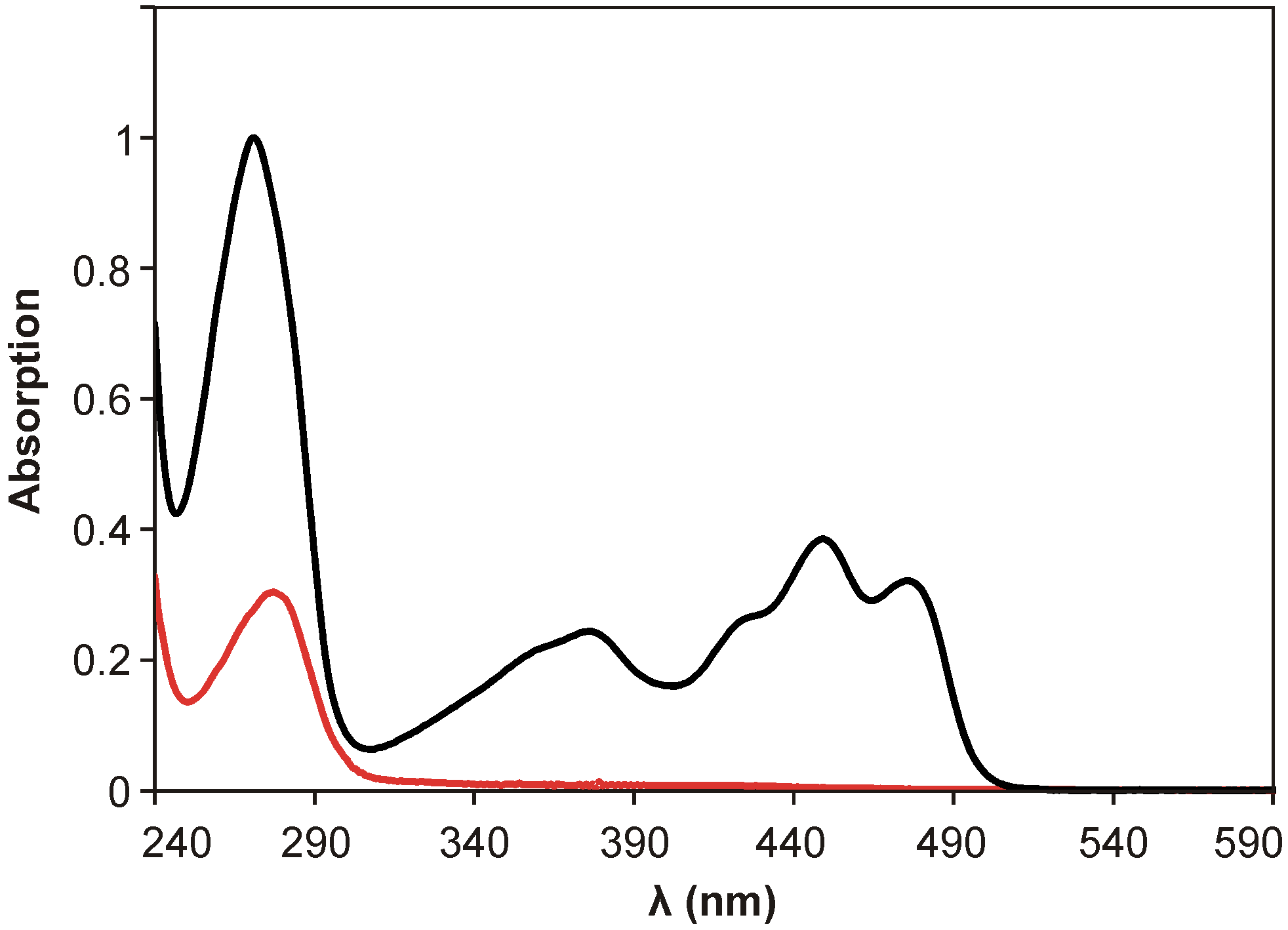

Supplement: Figure S2 — Absorption spectrum of YLOV and Apo-YLOV. Spectra were derived from 30 µM heterologously expressed dark state YLOV before (black line) and after deflavination (red line). Absence of the typical flavin absorbance between 310 nm and 510 nm indicates complete chromophore removal. The spectra were measured using a DU-520 spectrophotometer (Beckman-Coulter). (TIF) [file pone.0081268.s002.tif]

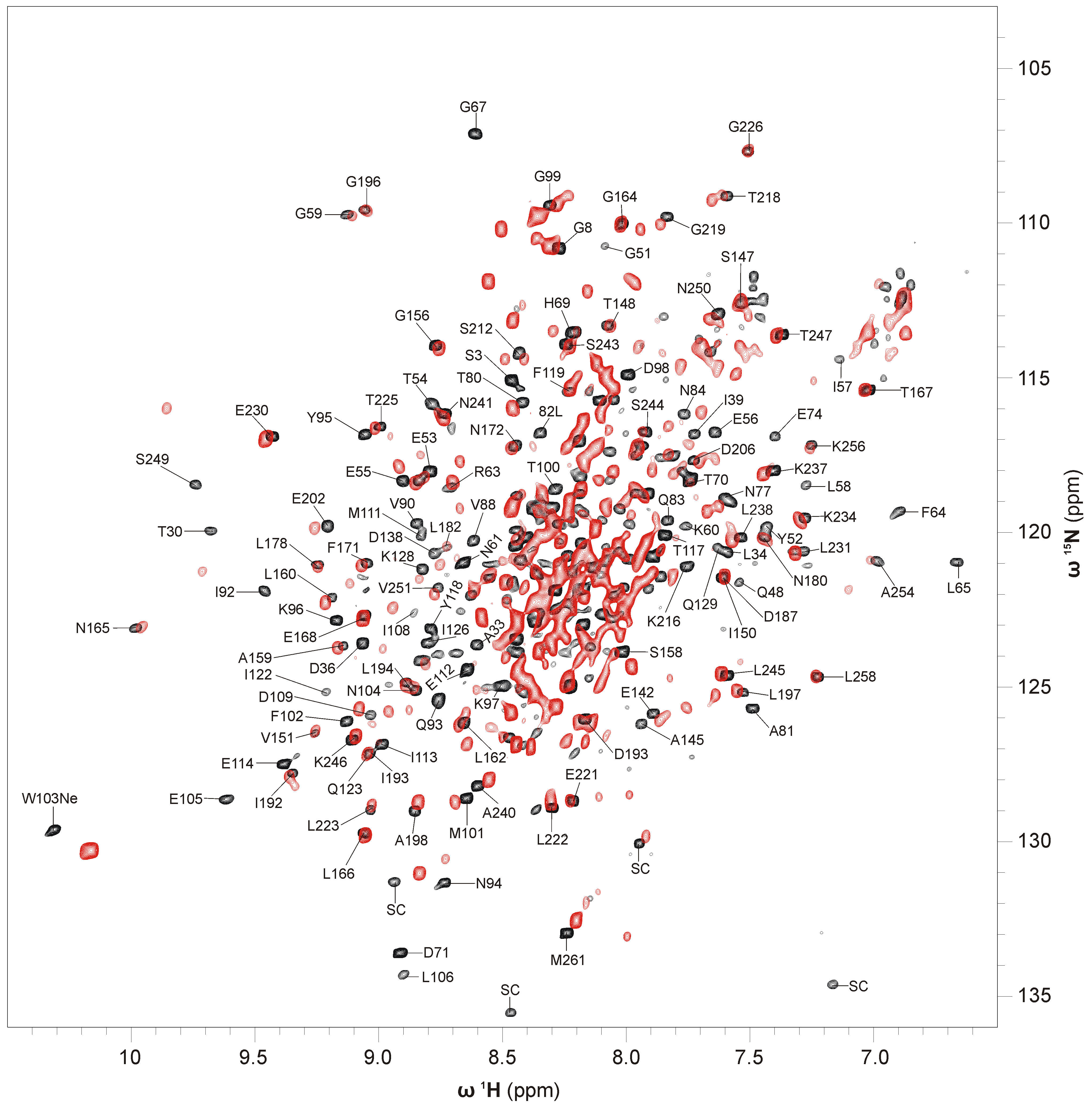

Supplement: Figure S3 — Superposition of 1H-15N-TROSY spectra of native and apo form of YtvA. 1H-15N-TROSY spectra of native and deflavinated YtvA are shown in black and red, respectively. Both samples were uniformly 2H-15N-labeled. Sequentially assigned amide resonances of YtvA outside the central region of the spectrum are labeled with residue type and sequence position (SC: resonances most likely belonging to side chains). See main text for details. (TIF) [file pone.0081268.s003.tif]

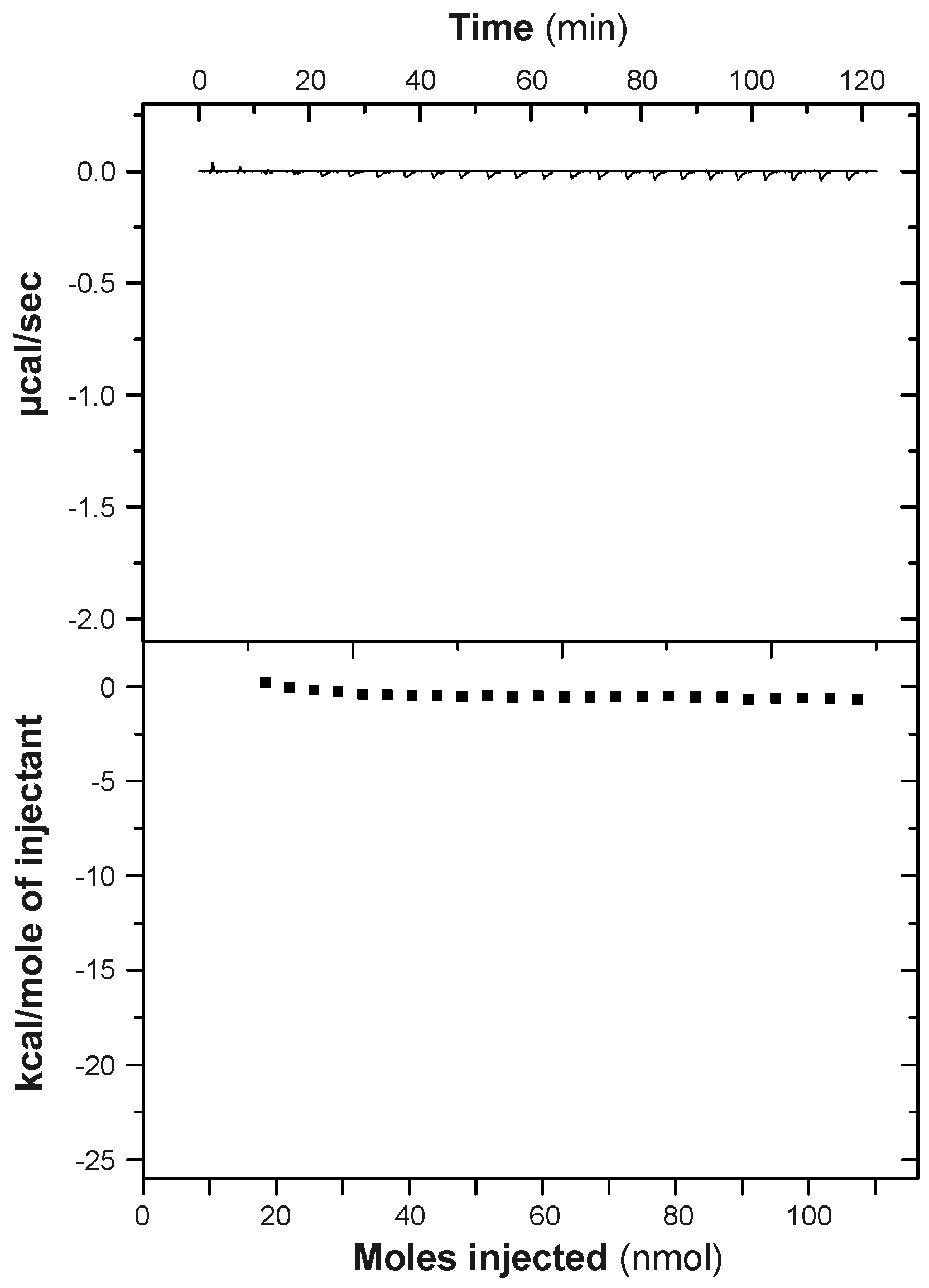

Supplement: Figure S4 — ITC thermogram of the titration of Apo-YLOV into buffer. The upper panel displays the ITC thermogram derived from the titration of 400 µM Apo-YLOV into buffer. The heat generated by each injection is shown below. To illustrate the negligible dilution effects, the vertical axes were scaled to the same range used for evaluation of titrations performed with flavins. The titration experiment was performed at 25°C using a Microcal VP-ITC microcalorimeter. (TIF) [file pone.0081268.s004.tif]

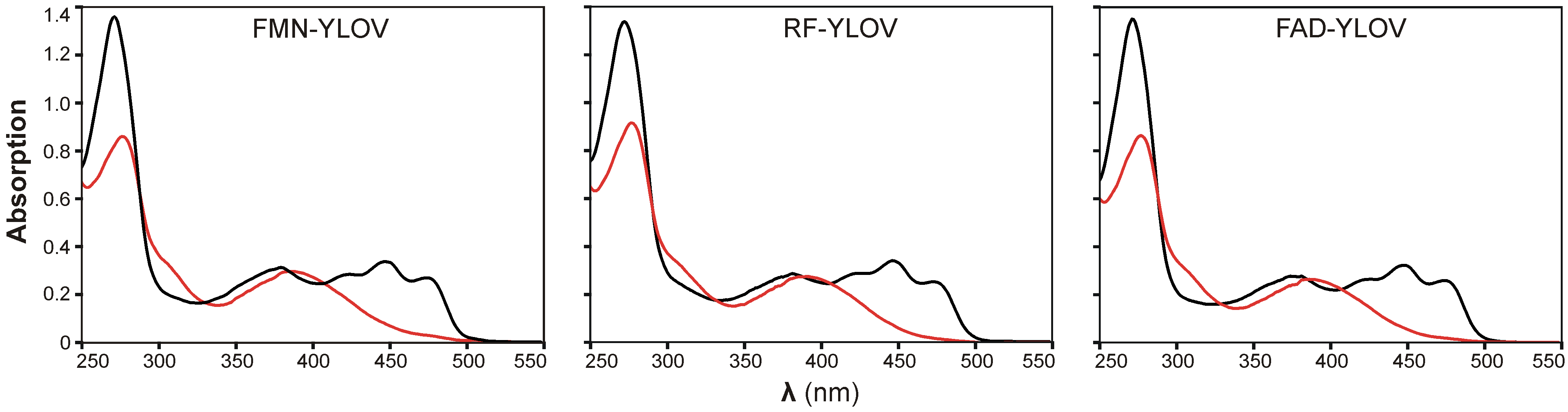

Supplement: Figure S5 — UV-Vis spectra of unexposed and illuminated YLOV reconstituted with flavins. The spectra were obtained from YLOV reconstituted with FMN, RF or FAD during ITC experiments. Spectra of unexposed and illuminated proteins are shown in black and red, respectively. Absence of the typical chromophore absorbance pattern at λ > 410 nm after illumination with blue-light indicates formation of the covalent adduct between carbon C4a of the isoalloxazine ring and the sulfur within the side chain of cysteine C62 proving that all reconstituted variants were photochemically active. Photo conversion and accumulation of the photoactivated state were achieved by illuminating the samples contained in a 200 µl pipette tip for 30 s with a high power LED (H6-RGB-9; λmax = 460 nm; Φ = 430 mW; Roithner Lasertechnik; Austria). The spectra were measured using a DU-520 spectrophotometer (Beckman-Coulter). (TIF) [file pone.0081268.s005.tif]

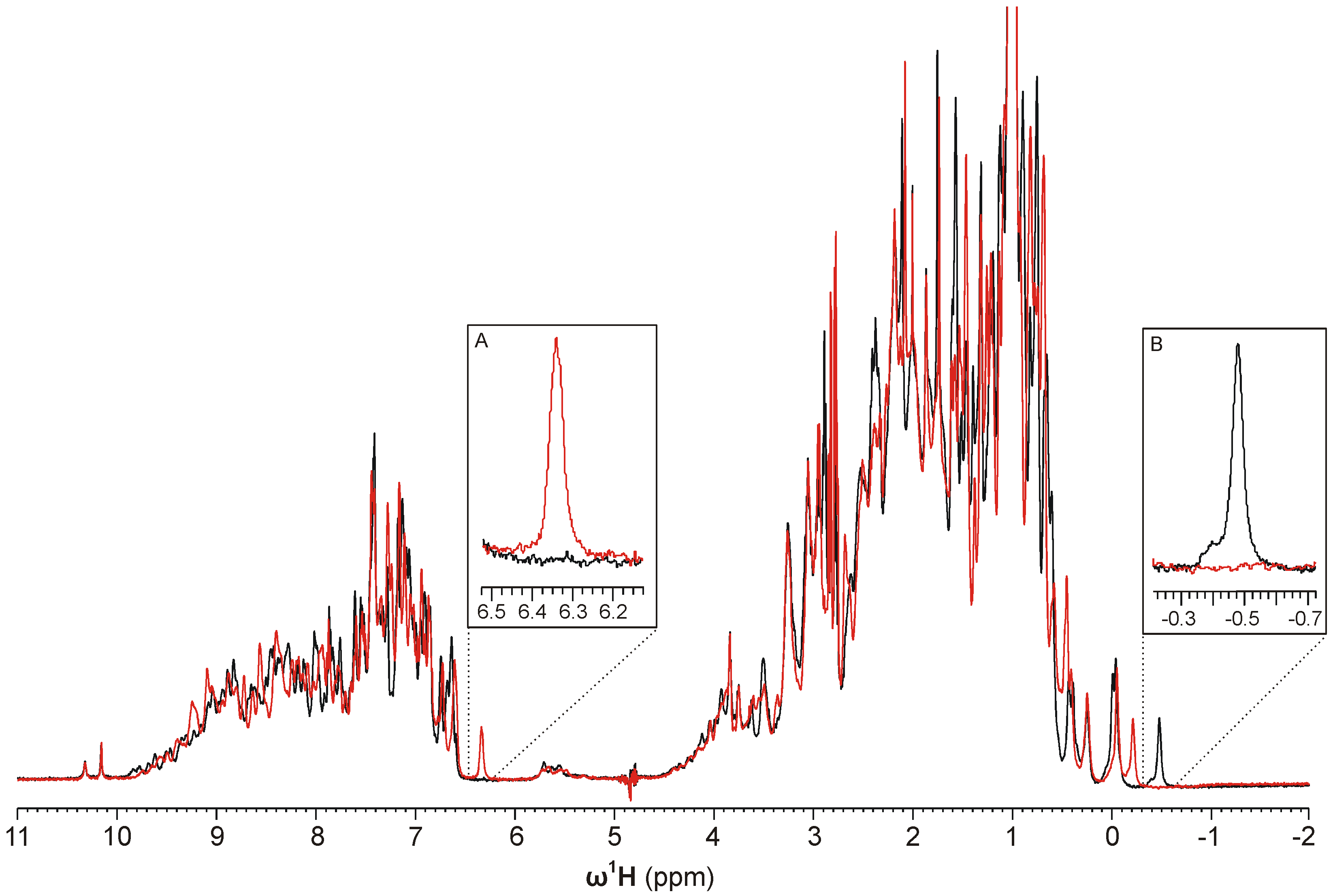

Supplement: Figure S6 — Superimposed 1H-NMR spectra of dark-state and illuminated YLOV. The 1H-spectra of dark-state (black) and illuminated YLOV (red) differ significantly indicating light-induced structural rearrangements. The signals enlarged in the insets were used to monitor the dark-state recovery kinetics by tracking its intensity changes during conversion of photo activated YLOV to the ground state. The intensity of the signal at -0.475 ppm (B) is maximum in the ground state going to zero after illumination whereas the signal at 6.46 ppm (A) displays opposing characteristics. Corresponding signals are also present in 1H-spectra of YtvA and YLOV variants containing RF or FAD. The signal at -0.475 ppm belongs to a methyl group of I108. The origin of the other signal (B) is unknown. To obtain the lit-state spectrum, sample was illuminated using our previously described fiber coupled light source [50]. Spectra were acquired at 27°C on a 600 MHz NMR spectrometer (Bruker Avance III) equipped with a 5 mm triple resonance PFG (z-axis) cryo probe head. (TIF) [file pone.0081268.s006.tif]

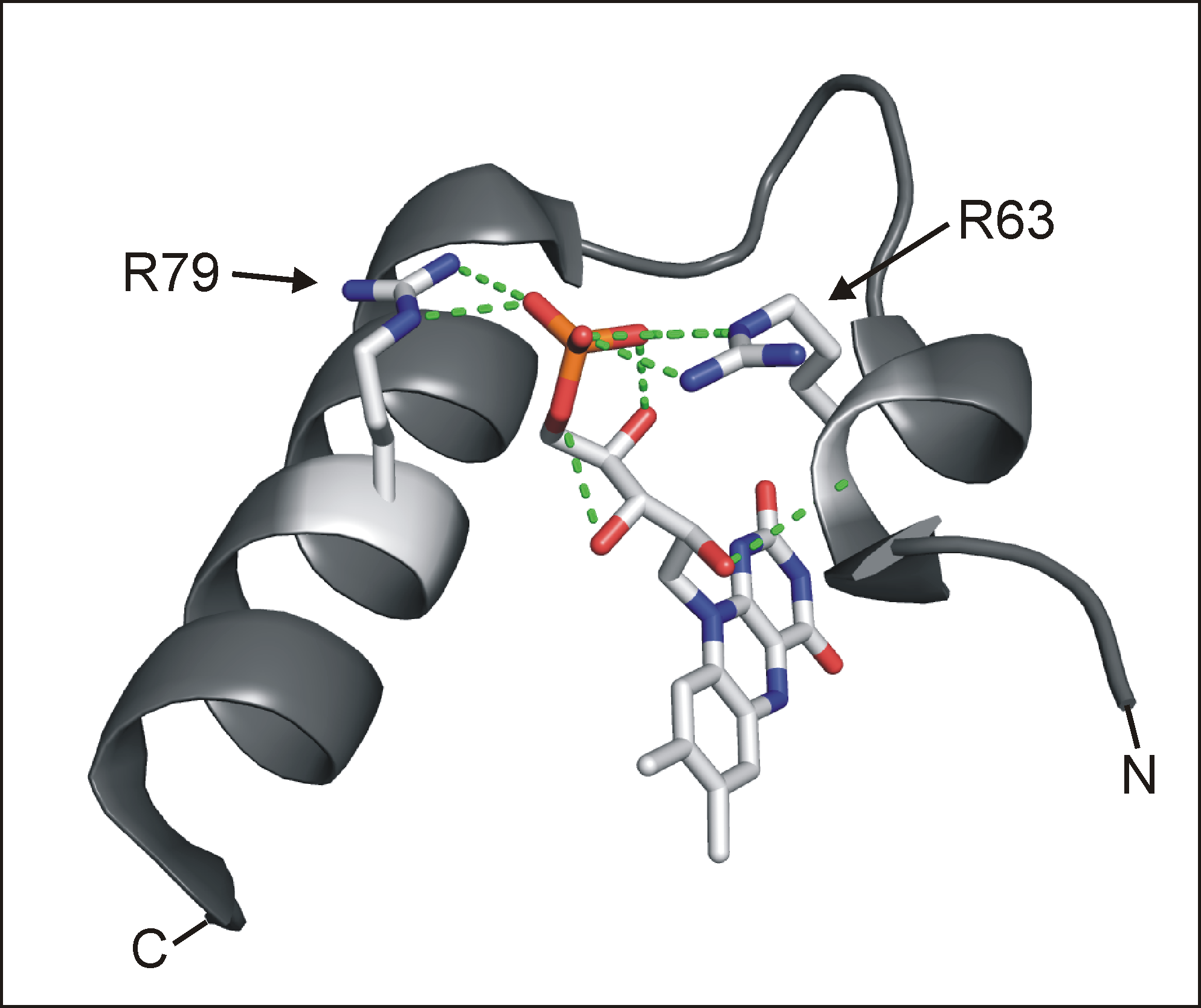

Supplement: Figure S7 — Polar interactions between FMN and arginines R63 and R79 of the YtvA LOV domain. Close-up of the YtvA LOV domain structure (PDB-ID: 2PR5 [29]) showing polar interactions (green dotted lines) between the phosphate group of FMN and the side chains of arginines R63 and R79. Heteroatoms are color-coded: nitrogen (blue), oxygen (red), phosphor (orange). (TIF) [file pone.0081268.s007.tif]

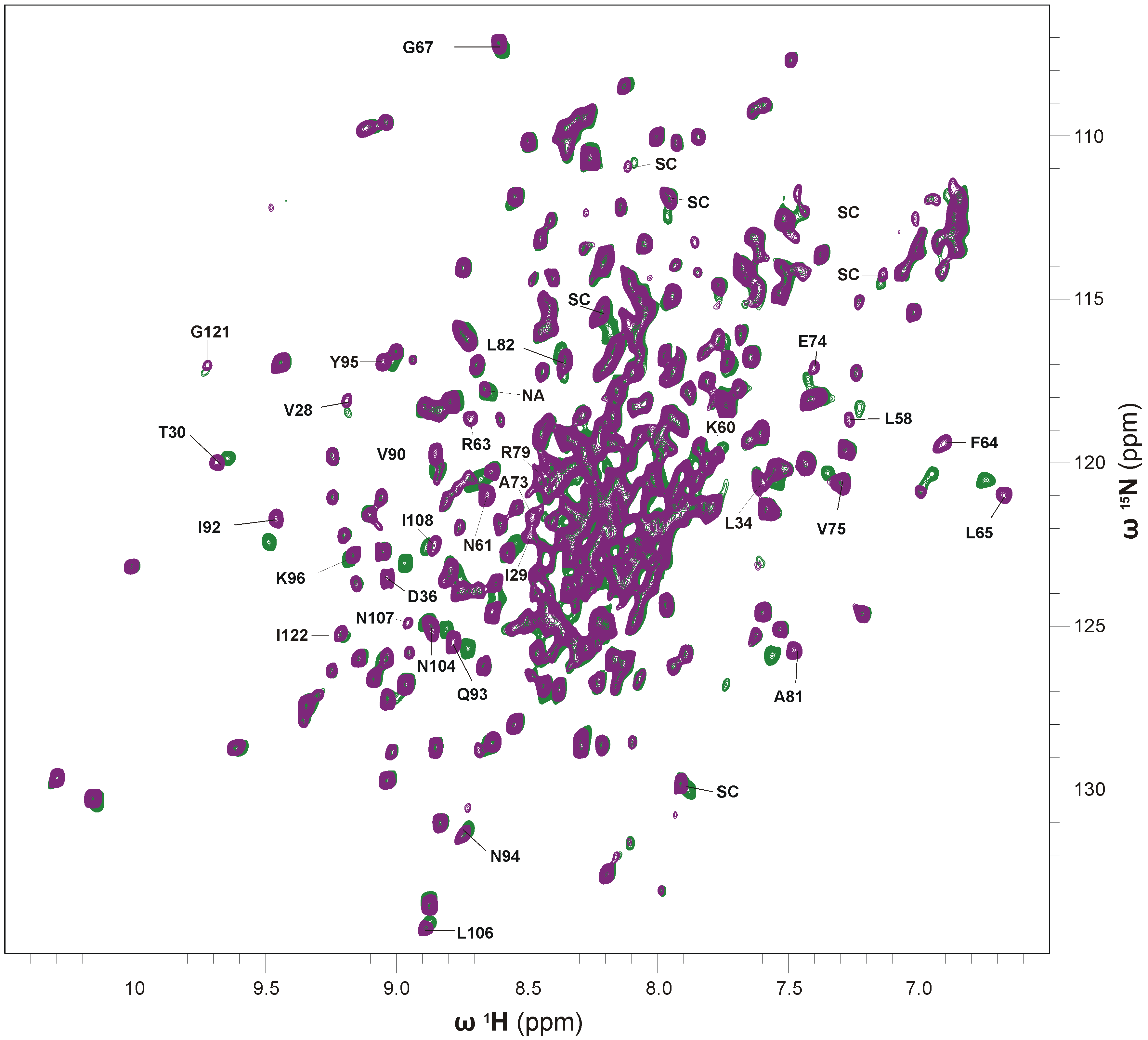

Supplement: Figure S8 — Superimposed 1H-15N-TROSY spectra of YtvA reconstituted with FMN and RF. The 1H-15N-TROSY spectrum of YtvA reconstituted with FMN and RF is colored in magenta and green, respectively. Both samples were uniformly 2H-15N-labeled. Resonances belonging to amino acids significantly or slightly affected by exchange of FMN against RF are labeled with residue type and sequence position (SC: Unassigned amide resonances most-likely belonging to side-chains). See main text for details. Spectra were acquired at 27°C on a 600 MHz NMR spectrometer (Bruker Avance III) equipped with a 5 mm triple resonance PFG (z-axis) cryo probe head. (TIF) [file pone.0081268.s008.tif]
