# Supplementary material for: LOV Takes a Pick: Thermodynamic and Structural Aspects of the Flavin-LOV-Interaction of the Blue-Light Sensitive Photoreceptor YtvA from Bacillus subtilis
Source: PLoS One. 2013 Nov 21;8(11):e81268. doi: 10.1371/journal.pone.0081268 (PMC3836802; doi:10.1371/journal.pone.0081268)
Supplement: Protocol S1 — Exchange of RF by FMN in YtvA and YLOV under native conditions. (DOCX) [file pone.0081268.s009.docx]

**LOV takes a pick:** **thermodynamic and structural aspects of the Flavin-LOV-Interaction of the blue-light sensitive Photoreceptor YtvA from *Bacillus subtilis***

**Supplementary Material**

**Protocol S1**

**Protocol S1**

**Exchange of RF by FMN in YtvA and YLOV under native conditions:**

Riboflavin incorporated into YtvA and YLOV during overexpression was exchanged against FMN by incubation of the proteins (c = 100 - 300 µM) in presence of 100-fold molar excess of FMN for at least 24 h at room temperature in the dark. Excess flavins were subsequently removed by size exclusion chromatography using the conditions described in the main text. Chromophore exchange was checked by HPLC-based analysis of the protein associated flavins before and after the exchange procedure. Prior HPLC, flavins were released from dark-adapted proteins (100 µM protein in 100 µl sample volume) by heat denaturation (5 min at 95°C in the dark). After immediate cooling on ice, protein was precipitated with 400 µl acetone and the sample was subsequently centrifuged for 10 min at 10000 x g. The supernatant was dried by vacuum centrifugation and the resulting pellet dissolved in 50 µl H_2_O. Finally, 15 µl of this solution were analysed by HPLC using a Shimadzu LC-6A chromatography station equipped with a C18-Pyramid column (Nucleodur^®^; 5 mm diameter; 100 mm length). Elution was done with a linear acetonitrile gradient (0-80% in H_2_O) and detection was carried out at 280 nm and 447 nm. Retention times of RF and FMN were determined in separate calibration runs using stock solutions (10 µg/ml) of these flavins as reference.

**Figure S1:**


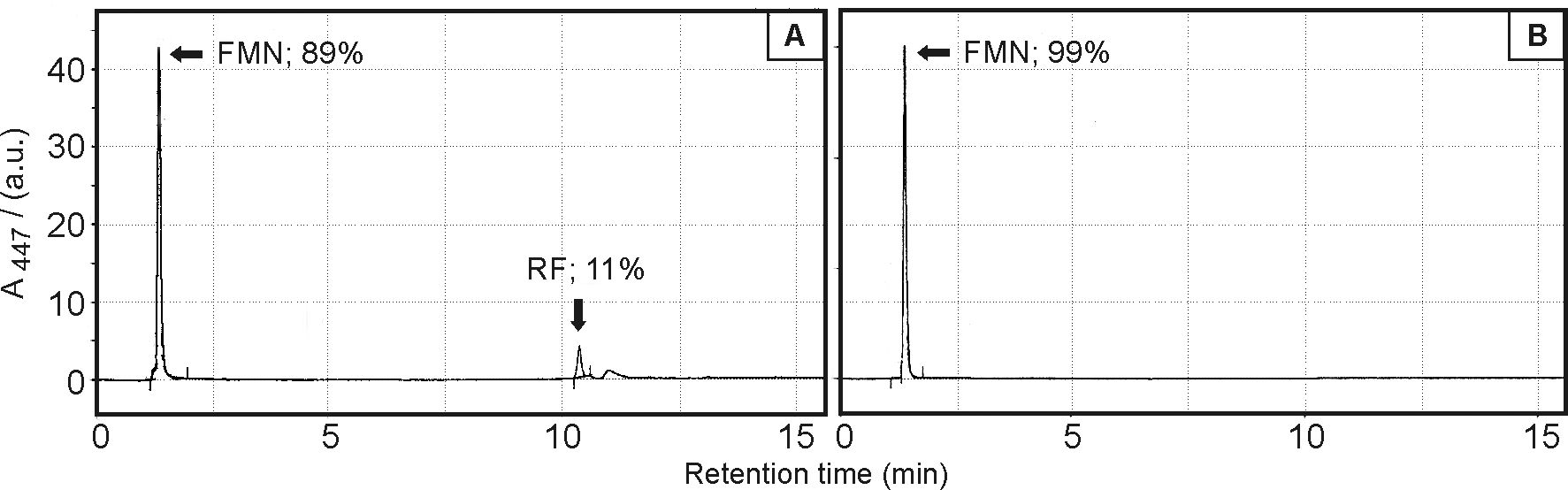


Figure S1: HPLC-based chromophore analysis of YLOV.

(A) Chromatogram of the flavins released from heterologously expressed YLOV proved the incorporation of 89% FMN and 11% riboflavin (RF) during overexpression in *E. coli*.
(B) After exchange of RF by FMN under native conditions (see text), heterologously expressed YLOV contained no detectable amount of RF. HPLC was performed using a Shimadzu LC-6A chromatography station equipped with a C18-Pyramid column (Nucleodur^®^; 5 mm diameter; 100 mm length). Elution was done with a linear acetonitrile gradient (0-80% in H_2_O) and detection was carried out at 280 nm and 447 nm.
