# Supplementary material for: LOV Takes a Pick: Thermodynamic and Structural Aspects of the Flavin-LOV-Interaction of the Blue-Light Sensitive Photoreceptor YtvA from Bacillus subtilis
Source: PLoS One. 2013 Nov 21;8(11):e81268. doi: 10.1371/journal.pone.0081268 (PMC3836802; doi:10.1371/journal.pone.0081268)
Supplement: Table S1 — Chemical shifts of YLOV reconstituted with FMN, RF and FAD. (PDF) [file pone.0081268.s010.pdf]

LOV takes a pick: thermodynamic and structural aspects of the Flavin-LOV-Interaction of the blue-light sensitive Photoreceptor YtvA from *Bacillus subtilis*

Supplementary Material

Table S1

Table S1: Chemical shifts of YLOV reconstituted with FMN, RF and FAD

Residue		FMN-YLOV		RF-YLOV		FMN vs. RF	FAD-YLOV		FMN vs. FAD	
No.	Type	ω ^1H_N (ppm)	ω ^{15}N (ppm)	ω ^1H_N (ppm)	ω ^{15}N (ppm)	CSD[1] (ppm)	ω ^1H_N (ppm)	ω ^{15}N (ppm)	CSD[1] (ppm)	Intensity ratio[2] (%)
1	Gly	-	-	-	-	-	-	-	-	-
2	Ala	-	-	-	-	-	-	-	-	-
3	Ser	-	-	-	-	-	-	-	-	-
4	Phe	-	-	-	-	-	-	-	-	-
5	Gln	-	-	-	-	-	-	-	-	-
6	Ser	-	-	-	-	-	-	-	-	-
7	Phe	8,066	124,12	8,065	124,04	0,008	-	-	-	disappeared
8	Gly	8,230	111,13	8,233	111,05	0,009	8,241	111,07	0,013	25,0
9	Ile	8,279	121,33	8,278	121,39	0,006	8,269	121,53	0,023	53,7
10	Pro	-	-	-	-	-	-	-	-	-
11	Gly	8,139	108,95	-	-	-	-	-	-	disappeared
12	Gln	7,862	120,91	7,871	120,94	0,009	-	-	-	disappeared
13	Leu	8,377	119,33	8,377	119,35	0,002	8,369	119,34	0,008	14,6
14	Glu	8,016	117,03	8,004	117,10	0,013	-	-	-	disappeared
15	Val	7,469	119,46	7,489	119,40	0,020	-	-	-	disappeared
16	Ile	8,020	119,20	8,022	119,20	0,002	-	-	-	disappeared
17	Lys	-	-	-	-	-	-	-	-	-
18	Lys	-	-	-	-	-	-	-	-	-
19	Ala	8,441	123,30	8,439	123,32	0,003	8,441	123,47	0,017	66,8
20	Leu	7,956	120,88	7,953	120,92	0,005	7,943	120,81	0,015	91,7
21	Asp	-	-	-	-	-	-	-	-	-
22	His	-	-	-	-	-	-	-	-	-
23	Val	-	-	-	-	-	-	-	-	-
24	Arg	-	-	-	-	-	-	-	-	-
25	Val	-	-	-	-	-	-	-	-	-
26	Gly	9,324	111,46	9,257	111,27	0,070	9,337	111,50	0,014	49,0
27	Val	-	-	-	-	-	-	-	-	-
28	Val	9,158	118,66	9,152	119,07	0,041	-	-	-	disappeared
29	Ile	8,483	122,58	8,504	122,77	0,028	8,507	122,51	0,024	33,7
30	Thr	9,630	120,16	9,593	120,08	0,038	9,632	120,26	0,011	30,4
31	Asp	7,362	118,18	7,364	118,14	0,004	7,394	118,24	0,032	26,5
32	Pro	-	-	-	-	-	-	-	-	-
33	Ala	8,631	124,05	8,619	124,17	0,017	8,627	124,04	0,004	14,6
34	Leu	7,585	120,86	7,571	120,82	0,014	7,588	120,86	0,003	26,3
35	Glu	8,294	122,22	-	-	-	-	-	-	-
36	Asp	9,046	123,73	8,994	123,19	0,074	9,053	123,84	0,013	28,3
37	Asn	8,111	115,71	8,169	115,84	0,060	8,125	115,74	0,015	18,8
38	Pro	-	-	-	-	-	-	-	-	-
39	Ile	7,731	117,09	7,701	117,09	0,030	-	-	-	disappeared
40	Val	9,379	120,58	9,358	120,54	0,022	-	-	-	disappeared
41	Tyr	7,611	123,33	7,609	123,32	0,003	7,617	123,30	0,007	20,3
42	Val	7,065	123,75	7,090	123,55	0,031	-	-	-	disappeared

Residue		FMN-YLOV		RF-YLOV		FMN vs. RF	FAD-YLOV		FMN vs. FAD	
No.	Type	ω $^{1}H_{N}$ (ppm)	ω ^{15}N (ppm)	ω $^{1}H_{N}$ (ppm)	ω ^{15}N (ppm)	CSD[1] (ppm)	ω $^{1}H_{N}$ (ppm)	ω ^{15}N (ppm)	CSD[1] (ppm)	Intensity ratio[2] (%)
43	Asn	8,308	123,64	8,308	123,64	0,000	8,308	123,64	0,000	74,0
44	Gln	8,937	118,63	-	-	-	-	-	-	-
45	Gly	-	-	-	-	-	-	-	-	-
46	Phe	8,033	123,38	8,042	123,38	0,009	-	-	-	disappeared
47	Val	7,289	124,73	7,305	124,75	0,016	7,304	124,74	0,015	27,8
48	Gln	8,458	119,41	8,465	119,40	0,007	8,468	119,41	0,010	25,2
49	Met	7,836	117,99	7,839	118,03	0,005	7,833	118,07	0,008	24,8
50	Thr	7,637	103,63	7,633	103,67	0,005	-	-	-	disappeared
51	Gly	8,112	111,05	8,102	110,90	0,018	8,139	111,21	0,032	24,6
52	Tyr	7,413	119,99	7,423	119,96	0,010	7,426	120,04	0,014	18,6
53	Glu	8,794	118,36	8,786	118,27	0,012	8,775	118,45	0,021	17,3
54	Thr	8,788	116,28	8,776	116,30	0,012	8,792	116,32	0,006	22,1
55	Glu	8,952	118,71	-	-	-	-	-	-	-
56	Glu	7,668	117,03	7,670	117,06	0,004	7,684	117,08	0,017	24,2
57	Ile	7,152	114,42	7,161	114,65	0,024	-	-	-	disappeared
58	Leu	7,260	118,61	7,234	118,32	0,039	-	-	-	disappeared
59	Gly	9,090	109,90	9,074	109,73	0,024	-	-	-	disappeared
60	Lys	7,722	119,93	7,691	119,69	0,039	7,681	119,87	0,041	35,4
61	Asn	8,693	121,47	8,734	121,10	0,055	8,695	121,34	0,013	21,0
62	Cys	8,103	123,27	8,064	123,33	0,039	-	-	-	disappeared
63	Arg	8,793	119,00	-	-	≥ 0,085	-	-	-	disappeared
64	Phe	6,978	120,10	-	-	≥ 0,140	7,013	120,12	0,035	22,6
65	Leu	6,745	121,26	-	-	≥ 0,080	-	-	-	disappeared
66	Gln	7,774	118,42	-	-	-	-	-	-	-
67	Gly	8,606	107,46	8,600	107,57	0,012	-	-	-	disappeared
68	Lys	8,231	123,62	-	-	≥ 0,072	-	-	-	disappeared
69	His	8,242	114,19	8,258	114,34	0,022	8,261	113,90	0,034	34,4
70	Thr	7,759	118,41	-	-	-	-	-	-	-
71	Asp	8,907	133,79	8,906	133,63	0,016	8,923	133,74	0,017	29,1
72	Pro	-	-	-	-	-	-	-	-	-
73	Ala	8,508	121,91	-	-	≥ 0,106	8,512	122,01	0,011	29,4
74	Glu	7,393	117,33	7,419	117,64	0,041	7,409	117,37	0,017	27,5
75	Val	7,346	120,98	7,351	120,80	0,018	7,351	121,00	0,006	38,1
76	Asp	8,242	121,01	-	-	≥ 0,051	8,250	121,04	0,008	25,4
77	Asn	7,620	119,32	-	-	≥ 0,081	7,628	119,32	0,008	31,2
78	Ile	7,887	121,62	-	-	≥ 0,037	7,888	121,63	0,001	24,9
79	Arg	8,472	120,26	-	-	≥ 0,159	8,467	120,25	0,005	32,0
80	Thr	8,428	115,90	8,431	115,90	0,003	8,430	115,85	0,006	24,8
81	Ala	7,480	126,03	7,564	126,11	0,084	7,482	126,07	0,005	24,6
82	Leu	8,368	117,07	8,425	117,10	0,057	8,375	117,15	0,011	25,9
83	Gln	7,877	119,86	7,847	119,88	0,031	7,895	119,88	0,018	20,7
84	Asn	7,772	116,50	7,738	116,62	0,036	7,778	116,49	0,006	23,3
85	Lys	8,199	120,50	8,218	120,29	0,028	8,210	120,51	0,010	30,1
86	Glu	8,431	120,49	8,414	120,50	0,017	8,440	120,47	0,009	34,9

Residue		FMN-YLOV		RF-YLOV		FMN vs. RF	FAD-YLOV		FMN vs. FAD	
No.	Type	ω ^1H_N (ppm)	ω ^{15}N (ppm)	ω ^1H_N (ppm)	ω ^{15}N (ppm)	CSD[1] (ppm)	ω ^1H_N (ppm)	ω ^{15}N (ppm)	CSD[1] (ppm)	Intensity ratio[2] (%)
87	Pro	-	-	-	-	-	-	-	-	-
88	Val	8,663	120,50	8,668	120,48	0,005	8,671	120,53	0,008	22,6
89	Thr	8,184	125,34	8,241	125,37	0,058	8,206	125,42	0,024	36,3
90	Val	8,896	120,19	8,901	120,72	0,052	8,915	120,21	0,019	29,4
91	Gln	8,372	121,15	8,348	121,42	0,036	8,367	121,13	0,006	29,2
92	Ile	9,369	122,55	9,396	123,24	0,074	9,384	122,64	0,017	38,1
93	Gln	8,838	126,31	8,782	126,57	0,062	8,833	126,29	0,006	32,9
94	Asn	8,836	132,19	8,833	132,07	0,013	8,845	132,16	0,010	40,3
95	Tyr	9,092	117,39	9,022	117,42	0,069	9,111	117,40	0,019	45,6
96	Lys	9,154	123,20	9,175	123,26	0,022	-	-	-	disappeared
97	Lys	8,459	125,03	8,446	125,25	0,025	8,484	125,03	0,025	30,7
98	Asp	7,969	115,13	7,974	115,17	0,007	7,963	115,13	0,006	17,9
99	Gly	8,315	109,62	8,325	109,71	0,014	8,326	109,63	0,010	20,3
100	Thr	8,280	118,61	8,285	118,57	0,006	8,290	118,54	0,011	15,8
101	Met	8,658	128,82	8,667	128,84	0,009	8,669	128,82	0,011	30,2
102	Phe	9,233	126,27	9,215	126,10	0,024	9,231	126,19	0,009	23,7
103	Trp	8,885	124,81	8,893	124,82	0,008	8,883	124,84	0,003	28,7
104	Asn	8,756	125,29	8,711	125,26	0,046	8,778	125,34	0,022	34,7
105	Glu	9,472	128,67	9,461	128,70	0,011	9,474	128,64	0,004	29,3
106	Leu	8,897	134,44	8,890	134,29	0,017	-	-	-	disappeared
107	Asn	8,944	125,25	8,907	125,19	0,038	8,961	125,32	0,018	31,9
108	Ile	8,871	122,93	8,908	123,05	0,039	8,877	122,82	0,013	22,4
109	Asp	9,037	126,30	9,067	126,40	0,032	9,075	126,17	0,040	24,7
110	Pro	-	-	-	-	-	-	-	-	-
111	Met	8,835	120,36	8,826	120,29	0,011	8,817	120,20	0,024	22,5
112	Glu	8,675	124,76	8,675	124,73	0,003	8,649	124,77	0,026	27,7
113	Ile	8,955	127,12	8,957	127,08	0,004	8,979	127,18	0,024	17,9
114	Glu	9,347	127,64	9,341	127,62	0,006	9,353	127,64	0,006	25,6
115	Asp	8,714	117,53	8,715	117,54	0,001	8,713	117,60	0,008	34,0
116	Lys	8,148	122,81	8,152	122,80	0,004	8,165	122,86	0,017	32,2
117	Thr	7,840	120,45	7,846	120,53	0,011	7,869	120,52	0,030	30,6
118	Tyr	8,827	123,50	8,826	123,50	0,001	8,828	123,50	0,001	25,5
119	Phe	8,222	115,84	8,228	115,64	0,020	8,213	115,83	0,009	23,5
120	Val	9,109	121,80	9,128	121,78	0,019	-	-	-	disappeared
121	Gly	9,710	117,41	9,707	117,64	0,022	9,718	117,42	0,008	24,3
122	Ile	9,177	125,29	9,146	125,36	0,032	-	-	-	disappeared
123	Gln	9,033	128,06	8,975	127,76	0,066	9,059	128,05	0,026	46,6
124	Asn	8,307	122,23	-	-	-	-	-	-	-
125	Asp	8,400	125,31	8,386	125,67	0,039	8,421	125,29	0,021	32,5
126	Ile	8,683	123,05	8,657	122,94	0,028	-	-	-	disappeared
127	Thr	8,743	121,83	8,745	121,82	0,002	8,750	121,88	0,008	18,0
128	Lys	8,144	131,74	8,141	131,70	0,004	8,165	131,74	0,021	27,6

[1]) Chemical shift differences (CSD) were calculated with CCPN v2.1.5 using a scaling factor of 0.1 for ^{15}N chemical shifts
[2]) Intensity ratios of corresponding cross peaks from FMN-YLOV and FAD-YLOV
